# Supplementary material for: Healthcare experiences of perpetrators of domestic violence and abuse: a systematic review and meta-synthesis
Source: BMJ Open. 2021 May 19;11(5):e043183. doi: 10.1136/bmjopen-2020-043183 (PMC8137202; doi:10.1136/bmjopen-2020-043183)
Supplement: Supplementary data [file bmjopen-2020-043183supp001.pdf]

Table 1 The eMERGe meta-ethnography reporting guidance

From: Improving reporting of meta-ethnography: the eMERGe reporting guidance

Study: Calcia MA, Bedi S, Lempp H, Howard LM, Oram S. The healthcare experiences of perpetrators of domestic violence and abuse: a systematic review and meta-synthesis

No.	Criteria Headings	Reporting Criteria
Phase 1—Selecting meta-ethnography and getting started		
Introduction		
1	Rationale and context for the meta-ethnography	Describe the gap in research or knowledge to be filled by the meta-ethnography, and the wider context of the meta-ethnography<br><br>**Introduction (pages 3-5)**
2	Aim(s) of the meta-ethnography	Describe the meta-ethnography aim(s)<br><br>**Introduction (page 5)**
3	Focus of the meta-ethnography	Describe the meta-ethnography review question(s) (or objectives)<br><br>**Introduction (page 5)**
4	Rationale for using meta-ethnography	Explain why meta-ethnography was considered the most appropriate qualitative synthesis methodology<br><br>**Methods – Data synthesis (pages 8-9)**
Phase 2—Deciding what is relevant		
Methods		
5	Search strategy	Describe the rationale for the literature search strategy

No.	Criteria Headings	Reporting Criteria
		**Methods – Search strategy (page 6-7)**
6	Search processes	Describe how the literature searching was carried out and by whom<br><br>**Methods (pages 6-7)**
7	Selecting primary studies	Describe the process of study screening and selection, and who was involved<br><br>**Methods – Selection process (page 8)**
Findings		
8	Outcome of study selection	Describe the results of study searches and screening<br><br>**Results (pages 10-12)**<br>**PRISMA diagram (figure 1)**
Phase 3—Reading included studies		
Methods		
9	Reading and data extraction approach	Describe the reading and data extraction method and processes<br><br>**Methods (pages 8-9)**
Findings		
10	Presenting characteristics of included studies	Describe characteristics of the included studies<br><br>**Results (pages 10-12)**<br>**Table 2 (page 11)**
Phase 4—Determining how studies are related		
Methods		

No.	Criteria Headings	Reporting Criteria
11	Process for determining how studies are related	Describe the methods and processes for determining how the included studies are related:<br>- Which aspects of studies were compared<br>AND<br>- How the studies were compared<br><br>**Results (pages 11-21)**
Findings		
12	Outcome of relating studies	Describe how studies relate to each other<br><br>**Results (pages 19-24)**
Phase 5—Translating studies into one another		
Methods		
13	Process of translating studies	Describe the methods of translation:<br>- Describe steps taken to preserve the context and meaning of the relationships between concepts within and across studies- Describe how the reciprocal and refutational translations were conducted- Describe how potential alternative interpretations or explanations were considered in the translations<br><br>**Results and Discussion (pages 19-24)**
Findings		
14	Outcome of translation	Describe the interpretive findings of the translation.<br><br>**Results and Discussion (pages 19-26)**
Phase 6—Synthesizing translations		
Methods		
15	Synthesis process	Describe the methods used to develop overarching concepts (“synthesised translations”)<br>Describe how potential alternative interpretations or explanations were considered in the synthesis<br><br>**Results (page 11) and Discussion (pages 19-26)**

No.	Criteria Headings	Reporting Criteria
Findings		
16	Outcome of synthesis process	Describe the new theory, conceptual framework, model, configuration, or interpretation of data developed from the synthesis<br><br>**Results (third-order constructs) and Discussion (pages 19-26)**
Phase 7—Expressing the synthesis		
Discussion		
17	Summary of findings	Summarize the main interpretive findings of the translation and synthesis and compare them to existing literature<br><br>**Discussion (pages 25-29)**
18	Strengths, limitations, and reflexivity	Reflect on and describe the strengths and limitations of the synthesis:<br>- Methodological aspects—for example, describe how the synthesis findings were influenced by the nature of the included studies and how the meta-ethnography was conducted.- Reflexivity—for example, the impact of the research team on the synthesis findings<br><br>**Discussion, sub-section Strengths and Limitations (pages 27-28)**<br>**Reflexivity (pages 9-10)**
19	Recommendations and conclusions	Describe the implications of the synthesis<br><br>**Discussion (pages 25-29) and Conclusion (page 29)**
