# Supplementary material for: Healthcare experiences of perpetrators of domestic violence and abuse: a systematic review and meta-synthesis
Source: BMJ Open. 2021 May 19;11(5):e043183. doi: 10.1136/bmjopen-2020-043183 (PMC8137202; doi:10.1136/bmjopen-2020-043183)
Supplement: Supplementary data [file bmjopen-2020-043183supp002.pdf]

Appendix 2: Quality assessment

Study: Calcia MA, Bedi S, Lempp H, Howard LM, Oram S. The healthcare experiences of perpetrators of domestic violence and abuse: a systematic review and meta-synthesis

Assessment based on Harden A, Brunton G, Fletcher A, et al. Teenage pregnancy and social disadvantage: systematic review integrating controlled trials and qualitative studies. *Bmj* 2009;339

Vaan Rooij 2013: 8

Minor concerns over data collection (no mention of pilot interviews)
Minor concerns over analysis methods (no mention of strategies to ensure validity)
No concerns over how the study was conducted (e.g., aims/objectives were clear, sampling methods were clear and appropriate)

Overall assessment: medium

**Quality of reporting**	**Use of strategies to increase reliability and validity**	**Extent to which study findings reflected participants' perspectives and experiences**
Were the **aims and objectives** clearly reported? YES<br>Was there an adequate **description of the context** in which the research was carried out? YES<br>Was there an adequate description of the **sample** and the methods by which the sample was **identified and recruited**? YES<br>Was there an adequate description of the **methods used to collect data**? YES<br>Was there an adequate description of the **methods used to analyse data**? NOT CLEAR (has detailed description of analysis process but does not give name of type of qualitative method)	Were there attempts to establish the **reliability** of the data collection tools (for example, by use of interview topic guides)? YES<br>Were there attempts to establish the **validity** of the data collection tools (for example, with pilot interviews)? NOT CLEAR<br>Were there attempts to establish the **reliability of the data analysis methods** (for example, by use of independent coders)? YES<br>Were there attempts to establish the **validity of data analysis methods** (for example, by searching for negative cases)? NOT CLEAR	Did the study use **appropriate data collection methods** for helping participants to express their views? YES<br>Did the study use appropriate methods for ensuring the data analysis was grounded in the views of participants? YES<br>Did the study actively involve participants in its design and conduct? NOT CLEAR

Hester 2006 : 5

Minor concerns over data collection
Moderate concerns over analysis methods (method not clear)
Moderate concerns over how the study was conducted (sampling and recruitment methods not clear)

Overall assessment: low

**Quality of reporting**	**Use of strategies to increase reliability and validity**	**Extent to which study findings reflected participants' perspectives and experiences**
Were the aims and objectives clearly reported? YES<br>Was there an adequate description of the context in which the research was carried out? YES<br>Was there an adequate description of the sample and the methods by which the sample was identified and recruited? NO (not clear about how participants were approached or selected)<br>Was there an adequate description of the methods used to collect data? YES<br>Was there an adequate description of the methods used to analyse data? NO	Were there attempts to establish the reliability of the data collection tools (for example, by use of interview topic guides)? YES<br>Were there attempts to establish the validity of the data collection tools (for example, with pilot interviews)? NOT CLEAR<br>Were there attempts to establish the reliability of the data analysis methods (for example, by use of independent coders)? NO<br>Were there attempts to establish the validity of data analysis methods (for example, by searching for negative cases)? NO	Did the study use appropriate data collection methods for helping participants to express their views? YES<br>Did the study use appropriate methods for ensuring the data analysis was grounded in the views of participants? NOT CLEAR<br>Did the study actively involve participants in its design and conduct? NOT CLEAR

Hashimoto 2018: 7
Minor concerns over data collection (no mention of pilot interviews)
Minor concerns over data analysis
No concerns over how study was conducted (aims/objectives, sampling and recruitment were clear and appropriate)

Overall assessment: medium

**Quality of reporting**	**Use of strategies to increase reliability and validity**	**Extent to which study findings reflected participants' perspectives and experiences**
Were the aims and objectives clearly reported? YES<br>Was there an adequate description of the context in which the research was carried out? YES<br>Was there an adequate description of the sample and the methods by which the sample was identified and recruited? YES<br>Was there an adequate description of the methods used to collect data? YES<br>Was there an adequate description of the methods used to analyse data? YES	Were there attempts to establish the reliability of the data collection tools (for example, by use of interview topic guides)? YES<br>Were there attempts to establish the validity of the data collection tools (for example, with pilot interviews)? NOT CLEAR<br>Were there attempts to establish the reliability of the data analysis methods (for example, by use of independent coders)? NOT CLEAR<br>Were there attempts to establish the validity of data analysis methods (for example, by searching for negative cases)? NOT CLEAR	Did the study use appropriate data collection methods for helping participants to express their views? YES<br>Did the study use appropriate methods for ensuring the data analysis was grounded in the views of participants? NOT CLEAR<br>Did the study actively involve participants in its design and conduct? NOT CLEAR

Morgan 2014: 9

Minor concerns over data collection (no mention of pilot interviews)
Minor concerns over data analysis (no mention of strategies to ensure validity)
No concerns over how study was conducted (aims/objectives, sampling and recruitment were clear and appropriate)

Overall assessment: medium

**Quality of reporting**	**Use of strategies to increase reliability and validity**	**Extent to which study findings reflected participants' perspectives and experiences**
Were the aims and objectives clearly reported? YES<br>Was there an adequate description of the context in which the research was carried out? YES<br>Was there an adequate description of the sample and the methods by which the sample was identified and recruited? YES<br>Was there an adequate description of the methods used to collect data? YES<br>Was there an adequate description of the methods used to analyse data? YES	Were there attempts to establish the reliability of the data collection tools (for example, by use of interview topic guides)? YES<br>Were there attempts to establish the validity of the data collection tools (for example, with pilot interviews)? NOT CLEAR<br>Were there attempts to establish the reliability of the data analysis methods (for example, by use of independent coders)? YES<br>Were there attempts to establish the validity of data analysis methods (for example, by searching for negative cases)? NOT CLEAR	Did the study use appropriate data collection methods for helping participants to express their views? YES<br>Did the study use appropriate methods for ensuring the data analysis was grounded in the views of participants? YES<br>Did the study actively involve participants in its design and conduct? NOT CLEAR

Swogger 2016: 9

Minor concerns over data collection (no mention of pilot interviews)
Minor concerns over data analysis (no mention of strategies to ensure validity)
No concerns over how study was conducted (aims/objectives, sampling and recruitment were clear and appropriate)

Overall assessment: medium

**Quality of reporting**	**Use of strategies to increase reliability and validity**	**Extent to which study findings reflected participants' perspectives and experiences**
Were the aims and objectives clearly reported? YES<br>Was there an adequate description of the context in which the research was carried out? YES<br>Was there an adequate description of the sample and the methods by which the sample was identified and recruited? YES<br>Was there an adequate description of the methods used to collect data? YES<br>Was there an adequate description of the methods used to analyse data? YES	Were there attempts to establish the reliability of the data collection tools (for example, by use of interview topic guides)? YES<br>Were there attempts to establish the validity of the data collection tools (for example, with pilot interviews)? NOT CLEAR<br>Were there attempts to establish the reliability of the data analysis methods (for example, by use of independent coders)? YES<br>Were there attempts to establish the validity of data analysis methods (for example, by searching for negative cases)? NOT CLEAR	Did the study use appropriate data collection methods for helping participants to express their views? YES<br>Did the study use appropriate methods for ensuring the data analysis was grounded in the views of participants? YES<br>Did the study actively involve participants in its design and conduct? NOT CLEAR

Bacchus 2018: 7

Minor concerns over data collection (no mention of pilot interviews)
Moderate concerns over data analysis (no mention of strategies to ensure validity)
No concerns over how study was conducted (aims/objectives, sampling and recruitment were clear and appropriate)

Overall assessment: medium

**Quality of reporting**	**Use of strategies to increase reliability and validity**	**Extent to which study findings reflected participants' perspectives and experiences**
Were the aims and objectives clearly reported? YES<br>Was there an adequate description of the context in which the research was carried out? YES<br>Was there an adequate description of the sample and the methods by which the sample was identified and recruited? YES<br>Was there an adequate description of the methods used to collect data? YES<br>Was there an adequate description of the methods used to analyse data? NOT CLEAR	Were there attempts to establish the reliability of the data collection tools (for example, by use of interview topic guides)? YES<br>Were there attempts to establish the validity of the data collection tools (for example, with pilot interviews)? NOT CLEAR<br>Were there attempts to establish the reliability of the data analysis methods (for example, by use of independent coders)? YES<br>Were there attempts to establish the validity of data analysis methods (for example, by searching for negative cases)? NOT CLEAR	Did the study use appropriate data collection methods for helping participants to express their views? YES<br>Did the study use appropriate methods for ensuring the data analysis was grounded in the views of participants? NOT CLEAR<br>Did the study actively involve participants in its design and conduct? NO
