# Supplementary material for: Healthcare experiences of perpetrators of domestic violence and abuse: a systematic review and meta-synthesis
Source: BMJ Open. 2021 May 19;11(5):e043183. doi: 10.1136/bmjopen-2020-043183 (PMC8137202; doi:10.1136/bmjopen-2020-043183)
Supplement: Author's manuscript [file bmjopen-2020-043183.draft_revisions.pdf]

BMJ Open

BMJ Open is committed to open peer review. As part of this commitment we make the peer review history of every article we publish publicly available.

When an article is published we post the peer reviewers' comments and the authors' responses online. We also post the versions of the paper that were used during peer review. These are the versions that the peer review comments apply to.

The versions of the paper that follow are the versions that were submitted during the peer review process. They are not the versions of record or the final published versions. They should not be cited or distributed as the published version of this manuscript.

BMJ Open is an open access journal and the full, final, typeset and author-corrected version of record of the manuscript is available on our site with no access controls, subscription charges or pay-per-view fees (http://bmjopen.bmj.com).

If you have any questions on BMJ Open's open peer review process please email info.bmjopen@bmj.com

BMJ Open

The healthcare experiences of perpetrators of domestic violence and abuse: a systematic review and meta-synthesis

Journal:	*BMJ Open*
Manuscript ID	bmjopen-2020-043183
Article Type:	Original research
Date Submitted by the Author:	30-Jul-2020

Complete List of Authors:	Calcia, Marilia; King's College London, Section of Women's Mental Health, Department of Health Service and Population Research, Institute of Psychiatry, Psychology and Neuroscience<br>Bedi, Simran; King's College London, Section of Women's Mental Health, Department of Health Service and Population Research, Institue of Psychiatry, Psychology and Neuroscience<br>Howard, Louise; King's College London, Section of Women's Mental Health, Department of Health Service and Population Research, Institute of Psychiatry, Psychology and Neuroscience<br>Lempp, Heidi; King's College London, Centre for Rheumatic Diseases, Department of Inflammation Biology, Faculty of Life Sciences and Medicine<br>Oram, Sian; King's College London, Section of Women's Mental Health, Department of Health Service and Population Research, Institute of Psychiatry, Psychology and Neuroscience

Keywords:	MENTAL HEALTH, PRIMARY CARE, Substance misuse < PSYCHIATRY, Forensic psychiatry < PSYCHIATRY, QUALITATIVE RESEARCH, SEXUAL MEDICINE

SCHOLARONE™
Manuscripts

BMJ

I, the Submitting Author has the right to grant and does grant on behalf of all authors of the Work (as defined in the below author licence), an exclusive licence and/or a non-exclusive licence for contributions from authors who are: i) UK Crown employees; ii) where BMJ has agreed a CC-BY licence shall apply, and/or iii) in accordance with the terms applicable for US Federal Government officers or employees acting as part of their official duties; on a worldwide, perpetual, irrevocable, royalty-free basis to BMJ Publishing Group Ltd ("BMJ") its licensees and where the relevant Journal is co-owned by BMJ to the co-owners of the Journal, to publish the Work in this journal and any other BMJ products and to exploit all rights, as set out in our licence.

The Submitting Author accepts and understands that any supply made under these terms is made by BMJ to the Submitting Author unless you are acting as an employee on behalf of your employer or a postgraduate student of an affiliated institution which is paying any applicable article publishing charge ("APC") for Open Access articles. Where the Submitting Author wishes to make the Work available on an Open Access basis (and intends to pay the relevant APC), the terms of reuse of such Open Access shall be governed by a Creative Commons licence – details of these licences and which Creative Commons licence will apply to this Work are set out in our licence referred to above.

Other than as permitted in any relevant BMJ Author's Self Archiving Policies, I confirm this Work has not been accepted for publication elsewhere, is not being considered for publication elsewhere and does not duplicate material already published. I confirm all authors consent to publication of this Work and authorise the granting of this licence.

For peer review only

The healthcare experiences of perpetrators of domestic violence and abuse: a systematic review and meta-synthesis

Marilia A. Calcia, Simran Bedi, Louise Howard, Heidi Lempp, Sian Oram

PROSPERO registration id: CRD42017073818

Authors, affiliation and contact:

Dr Marilia A. Calcia, Doctoral student, Health Service and Population Research Department, Institute of Psychiatry, Psychology and Neuroscience (IoPPN), King's College London marilia.a.calcia@kcl.ac.uk
Simran Bedi, MBBS Student, KCL (Simran.bedi@kcl.ac.uk)
Prof Louise M Howard, Professor of Women's Mental Health, Health Service and Population Research Department, IoPPN, KCL (louise.howard@kcl.ac.uk)
Dr Heidi Lempp, Reader in Medical Sociology, Centre for Rheumatic Diseases, Department of Inflammation Biology, Faculty of Life Sciences and Medicine; King's College London KCL (heidi.lempp@kcl.ac.uk)
Dr Sian Oram, Senior Lecturer in Women's Mental Health and Head of the Section of Women's Mental Health, Health Service and Population Research Department, IoPPN, KCL (sian.oram@kcl.ac.uk)

Author contributions:

Dr Marilia A. Calcia: conceptualising the study; drafting and finalising review protocol; systematic literature search; primary reviewer for study selection and appraisal; meta-synthesis; wrote first draft of study for publication.

Simran Bedi: secondary reviewer for study selection and appraisal.

Professor Louise Howard, Dr Heidi Lempp, Dr Sian Oram: conceptualising the study; advising on methodology; supervision and editing of drafts.

Corresponding author:

Dr Marilia A. Calcia (marilia.a.calcia@kcl.ac.uk)

Conflict of interest:

For peer review only - http://bmjopen.bmj.com/site/about/guidelines.xhtml

All authors have completed the ICMJE uniform disclosure form at www.icmje.org/coi_disclosure.pdf and declare: Professor Louise Howard and Dr Sian Oram receive salary support from the UKRI Cross-disciplinary Network on Violence, Abuse and Mental Health (www.vamhn.co.uk). Professor Louise Howard also receives salary support from the NIHR SLAM/KCL Biomedical Research Centre and the NIHR South London Applied Research Collaboration. Dr Marilia A Calcia received salary support through a 3-month full time secondment to the Section of Women's Mental Health, Health Service and Population Research Department, IoPPN, KCL, from July-September 2018, from an NIHR Research Professorship to Louise Howard (NIHR-RP-R3-12-011). Dr Heidi Lempp, and Simran Bedi declare no financial relationships with any organisations that might have an interest in the submitted work in the previous three years. The authors declare no other relationships or activities that could appear to have influenced the submitted work.

Data statement:

Dr Marilia A. Calcia had full access to all the data in the study and takes responsibility for the integrity of the data and the accuracy of the data analysis.

Abstract

Objectives: Domestic violence and abuse (DVA) is highly prevalent, with severe adverse consequences to the health and wellbeing of survivors. There is a smaller evidence base on the health of DVA perpetrators and their engagement with healthcare services. This review examines the experiences of perpetrators of DVA of accessing healthcare services and the barriers and facilitators to their disclosure of abusive behaviours in these settings.

Design: A systematic review and meta-synthesis of qualitative studies.

Data sources: Qualitative studies published from the start of each database until May 2018, were identified in Cochrane, MEDLINE, EMBASE, PsycINFO, HMIC, BNID, CINAHL, ASSIA, IBSS, SSCI (peer-reviewed literature) and NDLTD, Open Grey and SCIE Online (grey literature). Eligibility criteria required studies used qualitative or mixed methods to report on the experiences of healthcare use by perpetrators of DVA. Meta-ethnographic methods were used to analyse the extracted data.

Results: Of 33,785 papers identified, 6 studies (n=122 participants; 121 men, 1 woman) met the inclusion criteria. Barriers to disclosure of DVA to healthcare staff included perpetrators' negative emotions and attitudes towards their abusive behaviours; fear of consequences of disclosure; and lack of trust in healthcare services' ability to address DVA. Facilitators of disclosure of DVA and engagement with healthcare services were

For peer review only - http://bmjopen.bmj.com/site/about/guidelines.xhtml

experiencing social consequences of abusive behaviours; feeling listened to by healthcare professionals; and offers of emotional and practical support for relationship problems by healthcare staff.

Conclusions: DVA perpetration is a complex issue with multiple barriers to healthcare engagement and disclosure. However, healthcare services can create positive conditions for the engagement of individuals who perpetrate abuse.

Article summary:

Strengths and limitations of this study**:**

- This study provides a systematic review of the healthcare experiences of domestic violence and abuse (DVA) perpetrators, including facilitators and barriers to disclosure of abusive behaviours to healthcare professionals
- Robust procedures for systematic reviewing and quality assessment were adopted, in line with Preferred Reporting Items for Systematic Reviews and Meta-Analyses reporting guidelines.
- The number of studies identified was small and some healthcare settings, e.g., emergency department, were not represented in the study sample.
- This review identified studies conducted in high-income countries and most participants were men who perpetrated intimate partner violence (IPV). The findings may, therefore, be less applicable to other settings such as low- and middle-income countries, or other populations such as perpetrators of family violence (FV) or female perpetrators of DVA.

Keywords: mental health; primary care; substance misuse; forensic psychiatry; sexual medicine; qualitative research

Word count: 5,043

INTRODUCTION

Domestic violence and abuse (DVA) are highly prevalent. In the year ending March 2018, an estimated two million people experienced domestic violence and abuse (DVA) in England and Wales alone[1]. Female victims outnumbered male victims approximately two to one. DVA is defined in the UK Domestic Abuse Bill 2020 as abusive behaviour (which may be physically, sexually, emotionally, psychologically or economically abusive or violent, threatening, controlling or coercive) by one person to another, when both are aged 16 or over and are “personally connected”. The term personally connected refers to two

For peer review only - http://bmjopen.bmj.com/site/about/guidelines.xhtml

people who are, or have been, in an intimate personal relationship, marriage or civil partnership, where each has parental responsibility to a child, or who are relatives[2].

DVA has a range of impacts on victims' health, including physical injury, chronic pain, gastrointestinal, and gynaecological problems, and depression, anxiety, and other mental disorders[3-5]. Children in families where DVA occurs have an increased risk of adverse behavioural, emotional and cognitive outcomes[6-8].

Less evidence exists regarding the health of DVA perpetrators, of which most relates to mental health. Systematic review evidence suggested both men and women with mental disorders are more likely to have a lifetime history of physical abuse towards a partner than those without a mental disorder, and the risk is higher in men than women[9]. However, few studies reported on recent violence (within the past year), and there was little data on whether violence occurred during acute episodes of mental illness or on the role of substance use as a confounder. A recent longitudinal study indicated that comorbid diagnoses of substance use disorders and personality disorders increase the risk of intimate partner violence perpetration against women in men with mental disorders[10].

It has been suggested that DVA perpetration is under-identified in both mental health and other healthcare settings. A study of over 300 service users cared for by community mental health teams in London showed that 1 in 10 of those individuals disclosed lifetime perpetration of DVA to the research team, but in only a third of those cases was their DVA perpetration known to mental health professionals[11]. Similarly, a medical records review of male patients in UK primary care showed that only 0.5% had DVA perpetration or victimisation documented in their medical records, despite 16% of the same patients disclosing negative behaviours in their relationship with a partner in a contemporaneous survey[12]. A systematic review has highlighted that mental health service users are in favour of routine enquiry of experiences of DVA by healthcare professionals and value when clinicians respond to disclosure by being non-discriminatory and supportive towards those who reported having experienced DVA[4]. To our knowledge no review has sought to synthesise evidence on DVA perpetrators' experiences of engaging with healthcare services or of the facilitators and barriers to disclosure of DVA perpetration to healthcare professionals.

This review aims to add to the evidence base by systematically identifying, reviewing, and synthesising qualitative studies that have explored the experiences of DVA perpetrators who access healthcare services. The review aimed to address the following questions:

(1) What are DVA perpetrators' experiences of being asked about and discussing DVA with healthcare staff?

For peer review only - http://bmjopen.bmj.com/site/about/guidelines.xhtml

(2) What are DVA perpetrators' experiences of using healthcare services when their history of DVA perpetration is known to healthcare staff?
(3) What are DVA perpetrators' experiences of seeking, accessing or being referred to for support to address their relationship difficulties and abusive behaviours by healthcare staff?
(4) What are DVA perpetrators' views on the association between their health and their relationship difficulties and abusive behaviours?

METHODS

We used the PRISMA reporting checklist when writing our report[13].

Inclusion criteria

Studies meeting the following criteria were included:

- Population: Adults (> 16 years of age) of any gender who are known to be, or identified themselves as, current or former perpetrators of DVA. DVA is defined as any incident, or pattern of incidents, of controlling, coercive or threatening behaviour, violence or abuse, between individuals 16 years or older who are, or have been, intimate partners or family members, regardless of gender or sexuality.
- Phenomenon of interest: Experiences of using healthcare services
- Setting: Healthcare services (primary care, emergency medicine, mental health services, secondary care medical or surgical specialties)
- Design: Any qualitative or mixed methods study design
- Type: Studies published in peer-reviewed journals, in the grey literature (ineligible formats listed under exclusion criteria), including masters' dissertations and doctoral theses
- Language of publication: English, Spanish, Portuguese or Italian
- Date of publication: Published from the start date of each database until 31 May 2018

Exclusion criteria:

- Design: Studies not using qualitative methods
- Phenomenon of interest: Studies where the published data were not sufficiently detailed to allow inclusion in the meta-synthesis, such as when participant accounts and/or researchers' interpretations were not clearly stated, or when data collection and analysis methods were not reported

For peer review only - http://bmjopen.bmj.com/site/about/guidelines.xhtml

- Setting: Studies where the data reported experiences of use of treatment programmes for DVA perpetrators ('perpetrator programmes'). Perpetrator programmes are interventions with specific aims that do not always involve healthcare services and have been studied separately from the wider healthcare literature[14]
- Type: Book chapters, conference papers, editorials, letters or general comment papers
- Language of publication: Studies published in languages other than English, Spanish, Italian or Portuguese

No geographic restrictions were set for this review.

Information sources

The following electronic bibliographic databases were searched: Cochrane, MEDLINE, EMBASE, PsycINFO, HMIC, BNID, CINAHL, ASSIA, IBSS, SSCI for peer-reviewed literature and NDLTD, OpenGrey and SCIE Online for grey literature.

Additionally, the primary reviewer conducted forwards citation tracking of included articles using Web of Science and Google Search.

Search strategy

Please see Table 1 for search terms. The databases were searched from their respective start dates to 15 March 2020.

Table 1. Search terms

DVA act - OR	Qualitative Research - OR	DVA perpetrator - OR
Intimate partner violence;<br>Intimate partner abuse; Partner abuse OR violence<br>Spouse abuse OR violence;<br>Battered wom*;<br>Couple* abuse OR violence;<br>Domestic abuse OR violence;<br>Family abuse OR violence;<br>Coercive control;<br>Control$ behavio$r;	Qualitative research/<br>Interview/<br>Theme$ or thematic<br>Qualitative study;<br>Qualitative<br>Ethnograph$;<br>Narrative;<br>Account;<br>Participant observation;<br>Grounded theory;<br>Interpretative;	Perpetrator$; Offender$;<br>Batterer$; Abuser$<br><br>**Setting:**<br>Patient$ OR<br>Service user$ OR<br>Consumer of<br>(mental)health care<br>Health$ OR<br>Healthcare

For peer review only - http://bmjopen.bmj.com/site/about/guidelines.xhtml

((abus$ OR batter$ OR violen$ OR beat$) adj2 (domestic OR partner$ OR family OR families OR spouse OR woman OR women OR men OR man OR female$ OR male$ OR wife OR wives OR husband$ OR boyfriend$ OR girlfriend$ OR elder$ OR brother$ OR sister$ OR father$ OR mother$ OR daughter$ OR son$)<br>(domestic adj2 homicid$).mp	Phenomenological Analysis;<br>Case stud$;<br>Focus group;<br>Framework analysis;<br>Framework approach;<br>Mixed-method$<br>Content analysis<br>Discourse analysis	Service$<br>Clinic$<br>Hospital$<br>Emergenc$<br>Outpatient

Patient and public involvement statement:

Patients or the public were not involved in the design, or conduct, or reporting, or dissemination plans of our research.

STUDY RECORDS

Data management:

Records were managed using EndNote software. Data were extracted into MS Excel.

Selection process:

The primary reviewer (author MC) conducted the searches and downloaded the results of each database into a separate file on a records management software (EndNote X9). A combined file was created at the end of the search and duplicate entries were removed.

The primary reviewer screened the studies' titles and abstracts and selected the studies that matched the inclusion criteria, followed by the screening of full-text records based on the inclusion criteria. The second reviewer checked the titles and abstracts of a random selection of 250 records and 25 full text records. Consensus about inclusion was reached for all papers following discussion between the reviewers. The number of studies excluded and the reasons for exclusion of full text studies is documented and reported in Figure 1.

Data collection process:

A data extraction form was developed by the primary reviewer (MC) and was piloted by the two reviewers with 4 studies. The pilot was refined until a final data extraction form

For peer review only - http://bmjopen.bmj.com/site/about/guidelines.xhtml

was agreed. The secondary reviewer performed an independent check of the data extraction in 2 of the 6 selected papers.

Basic data were extracted on study characteristics including sample size and characteristics; type of healthcare setting; and country. Using meta-ethnography methods, data were collected on first- and second-order constructs.

Data synthesis

Data were synthesised using meta-ethnography. Meta-ethnography treats the views of study participants as 'first-order constructs' and the explanations of each study's authors as 'second-order constructs'. The reviewers sought to identify and synthesise the first- and second- order constructs, which were similar across studies in a process called 'reciprocal translation'[15]. The relationships that reviewers identified between findings from the different primary studies were treated as 'third-order constructs'[15]. The reviewers looked for contradictory findings within and between studies (intra- and inter-study contradictions) via the process of refutational translation and analysed whether there were plausible explanations for these apparent contradictions, such as different contextual factors between studies[15,16].

Meta bias(es)

Risk of selection bias:

As described in the selection process, the reviewing team included a secondary reviewer, who conducted screening of a proportion of the search results separately. This method aimed to minimise the risk of bias arising from misclassification of studies as eligible/ineligible for inclusion in the review[17].

Risk of reporting bias:

The meta-ethnography methodology addresses risk of bias in the reporting of studies by separately considering the expressed views of participants and those of the study authors, as described in the Data Synthesis section.

Confidence in cumulative evidence

Risk of bias in individual studies

The studies included had their quality appraised by the primary reviewer, with the secondary reviewer providing an independent check of the quality assessment in 2 out of

For peer review only - http://bmjopen.bmj.com/site/about/guidelines.xhtml

the 6 selected papers. There was full agreement between the reviewers about the quality appraisal of the studies.

Quality assessment was based on Harden et al. (2009)[18] (see Table 2), who suggest 12 criteria based on the quality of reporting, use of strategies to increase reliability and validity, and the extent to which the findings reflected participants' views and experiences. Studies were classified as high quality (those meeting 10 or more criteria out of 12), medium quality (those meeting between seven and nine criteria) and low quality (those meeting fewer than seven criteria). Due to the current lack of consensus in the literature regarding quality assessment of qualitative studies[19,20], the quality assessment was not used to exclude studies from the review.

Table 2. Quality assessment (based on Harden et al. (2009)[18])

Quality of reporting	Use of strategies to increase reliability and validity	Extent to which study findings reflected participants' perspectives and experiences
Were the aims and objectives clearly reported?<br><br>Was there an adequate description of the context in which the research was carried out?<br><br>Was there an adequate description of the sample and the methods by which the sample was identified and recruited?<br><br>Was there an adequate description of the methods used to collect data?<br><br>Was there an adequate description of the	Were there attempts to establish the reliability of the data collection tools (for example, by use of interview topic guides)?<br><br>Were there attempts to establish the validity of the data collection tools (for example, with pilot interviews)?<br><br>Were there attempts to establish the reliability of the data analysis methods (for example, by use of independent coders)?<br><br>Were there attempts to establish the validity of	Did the study use appropriate data collection methods for helping participants to express their views?<br><br>Did the study use appropriate methods for ensuring the data analysis was grounded in the views of participants?<br><br>Did the study actively involve participants in its design and conduct?

For peer review only - http://bmjopen.bmj.com/site/about/guidelines.xhtml

methods used to analyse data?	data analysis methods (for example, by searching for negative cases)?	

RESULTS

A total of 229 abstracts were identified as potentially meeting the inclusion criteria during title and abstract screening. Following full-text screening (see Fig. 1), six studies were included in the review; four of these studies were published in peer-reviewed journals, one in a published report and one in a doctoral thesis.

Studies reported data from 122 participants, all but one of whom were adult males (see Table 3). All study participants were adults; other socio-demographic information, e.g., ethnicity, was not included in all papers. Five of the included studies focused on intimate partner violence (IPV); only Van Rooij et al. (2013)[21] included perpetrators of family violence (FV). Types of healthcare setting included general practice[22]; community addictions service[23]; sexual health clinic[24] and community forensic psychiatry[25]; two studies[25,26] explored participants' experiences of healthcare without specifying the setting. Half of the included studies (n=3) recruited participants from 'community', non-forensic settings[22-24], and three other studies recruited perpetrators of DVA from forensic settings, including perpetrator programmes[25], the criminal justice system[21] or prison[26]. For the latter group of studies, the constructs included in the analysis of this review related to experiences of use of healthcare services (including forensic psychiatry) but not of perpetrator programmes (see exclusion criteria). Four studies were conducted in the UK, one in the Netherlands[21] and one in North America[26].

All studies included individual qualitative interviews as the method of data collection; some studies also collected quantitative data, which are not reported in this review. The three studies that reported the qualitative analysis methods[21,22,26] each applied grounded theory[27]; the remaining three studies did not specify the data analytical methods used. The quality of reporting was assessed as medium in five of the six studies, and as low in one.

Table 3. Study characteristics

For peer review only - http://bmjopen.bmj.com/site/about/guidelines.xhtml

Study	Setting	Country	Sample size and characteristics	Community or forensic sample	Quality appraisal score
Hester et al. 2006[25]	Voluntary perpetrator programmes	UK	62 male IPV perpetrators	Mixed	Low (5/12)
Van Rooij et al 2013[21]*	Community forensic psychiatry	Netherlands	9 male and 1 female perpetrators of IPV and FV	Forensic	Medium (8/12)
Morgan et al 2014[22]*	General practice	UK	8 male IPV perpetrators	Community	Medium (9/12)
Swogger 2016[26]	Prison	US	15 male IPV perpetrators	Forensic	Medium (9/12)
Bacchus et al 2018[24]*	Sexual health clinic	UK	7 male IPV perpetrators	Community	Medium (7/12)
Hashimoto et al 2018[23]	Addiction services	UK	20 male IPV perpetrators	Community	Medium (7/12)

*these studies also included other participants who were not perpetrators of DVA. Data from those participants was not included in the analysis.

First order constructs

Analysis of the 6 qualitative papers identified 30 first order constructs, which were grouped into 6 key themes (see Table 4; participants' emotions towards DVA, participants' attitudes around DVA; their experiences of healthcare use; their triggers to wanting to instigate behavioural changes with regards to DVA perpetration; attitudes towards enquiry about DVA by healthcare services' staff; and concerns about confidentiality.

Emotions about DVA perpetration or relationship difficulties

Some participants described experiencing shame, embarrassment and regret over their abusive behaviours towards intimate partners and reported to researchers that those emotions meant they were unwilling to admit to those to clinicians[23]. Other participants reported feeling blamed by healthcare services when disclosing DVA perpetration,

For peer review only - http://bmjopen.bmj.com/site/about/guidelines.xhtml

particularly when they had also experienced DVA in their relationships and when the abuse led to the involvement of child protection services[26].

Attitudes towards DVA perpetration or relationship difficulties

Some participants minimized the importance of DVA, placing more emphasis on the positive aspects of their intimate relationship. Others normalised DVA, reporting views that abusive behaviours were an expected part of intimate relationships, rather than something that required specific help or intervention[23], indicating lack of awareness of what constitutes abuse in a relationship. Participants who reported that other issues in their lives (such as housing or financial problems) took priority over their relationship problems voiced that they were less likely to disclose relationship difficulties to healthcare professionals or to seek any type of support for those difficulties[21,23], reflecting a low prioritisation of DVA among other stressors.

Experiences of healthcare use

Some participants described how it took them many years to decide to seek help for DVA perpetration. Some experienced a long and convoluted route to access the support they wanted via healthcare services[23,25], while others did not seek help from healthcare professionals as they believed healthcare staff lacked the knowledge or expertise to address the perpetration of abusive behaviours[23]. When participants decided to seek help from healthcare professionals for relationship problems, some specifically wanted a referral for an 'anger management' service[23,25]. A range of different services were accessed, e.g., addiction, general practice and mental health services.

Experiences of psychological support were mixed, with some participants describing how individual or couples' therapy had been ineffective in reducing their use of abusive behaviours, or how they had felt blamed for their relationship problems within therapy[26]. Couples' counselling was perceived as unhelpful in changing perpetrators' behaviour when the intervention was focused on the victim's experiences rather than on the perpetrator's experiences and behaviours[26]. Other negative experiences mentioned by participants included when they thought that their general practitioners did not know enough about their personal life, for example about their sexuality, to allow disclosure of relationship problems[24].

Other participants, however, described how they were able to establish a close rapport with an individual healthcare worker who listened to them, or that they were pleased to gain access to psychotherapeutic interventions to focus on addressing their abusive behaviours[21,26]. Practical support, such as help with public transport fares to attend

For peer review only - http://bmjopen.bmj.com/site/about/guidelines.xhtml

appointments, was also appreciated. In one case, healthcare use led to access to professional reports that assisted in the participant's court case related to DVA[21]. Another attributed positive change to a combination of psychiatric treatment and a perpetrator programme undertaken while serving a prison sentence for DVA[26]; this is described in more detail in the following theme below.

Triggers that instigate behavioural changes with regards to DVA perpetration

Participants articulated how escalation of abuse, relationship breakdown, fear of losing contact with their children, being arrested or suffering other consequences of their behavior (such as being banned from social spaces) acted as important triggers to disclosing DVA perpetration and to seeking help via healthcare services[22,25]. Those events were consequences of perpetrating abuse, and some participants viewed them as 'wake-up calls' to come to terms with their behaviour and seek help. Engagement with mental health care and programmes for reduction of violent behaviour offered by prison healthcare services appeared to be a catalyst for change for one participant, who reported positive changes such as increased self-awareness and feeling less overwhelmed by his emotions through treatment[26].

Attitudes towards enquiry about DVA by healthcare services' staff

Participants' views about the acceptability of enquiry about DVA varied. Some thought that targeting enquiry only to individuals that show signs associated with DVA (e.g., physical marks or emotional distress) was more acceptable than universal enquiry in the primary care setting[22]; nevertheless, one participant (who had disclosed perpetration of physical, psychological and verbal abusive behaviours against a partner in the research questionnaire) was completely opposed to any questions about DVA, as he perceived DVA or relationship problems as an entirely private matter[22].

Those who were in favour of enquiry about DVA suggested that the initial assessment was the ideal time for enquiry, and that an explanation about the link between DVA and the health problems addressed by that service would help individuals understand the rationale for and feel more comfortable with the question. In the study conducted in a sexual health clinic, the provision of emotional support from a counsellor or non-medical health advisors was also mentioned as a condition for the acceptability of enquiry about DVA[24].

Concerns about confidentiality

Concerns about confidentiality acted as a key barrier to disclosure of relationship problems to clinicians; participants feared the potential consequences of disclosure, such as

For peer review only - http://bmjopen.bmj.com/site/about/guidelines.xhtml

involvement from children's social services[23,24]. One participant described that, for him, it was preferable to endure the consequences of being in a mutually abusive relationship than to seek help, due to his fear of losing the custody of his children if his relationship problems became known[23].

Having information about DVA documented in their general practice medical records was also a concern for some; other services, such as sexual health clinics, were perceived to offer more privacy and anonymity than primary care[24].

Table 4. First-order constructs

**Themes**	**First order constructs**
Emotions towards DVA perpetration	Shame, embarrassment, regret
Attitudes towards DVA perpetration	Normalisation, minimization, low prioritization of relationship problems
Experiences of healthcare use	Positive experiences:<br>good rapport with healthcare workers<br>feeling listened to<br>accessing interventions that address abusive behaviours<br>practical support<br><br>Negative experiences:<br>feeling blamed by healthcare services,<br>couples' therapy focusing on the victim,<br>delays and difficulties in accessing support,<br>believing that healthcare workers lack expertise on DVA or do not know enough about service users' personal lives<br><br>Referring to 'anger' as a way of disclosing DVA
Triggers to change	Escalation of abuse<br><br>Relationship breakdown<br><br>Fear of loss of contact with children

For peer review only - http://bmjopen.bmj.com/site/about/guidelines.xhtml

	Social consequences, including being arrested or being banned from social spaces<br><br>Positive engagement with healthcare services
Attitudes towards enquiry about relationship problems	Enquiry is more acceptable at the initial assessment of an individual by a healthcare service, and if it is linked with the healthcare problems for which that individual is seeking help<br><br>Availability of emotional support is a necessary element to allow enquiry about relationship problems
Concerns about confidentiality	Fear of children's social services' involvement<br><br>Perception that certain services (e.g., sexual health clinics) are more private than general practice

Second order constructs:

Thirty second order constructs were identified; these were grouped in three thematic categories (see Table 5). Six themes were duplications of the themes that emerged from each of the first order constructs (emotions towards DVA; attitudes around DVA; experiences of healthcare use; triggers to wanting to instigate behavioural changes with regards to DVA perpetration; attitudes towards enquiry about DVA by healthcare services' staff; and concerns about confidentiality); those will not be reported in this section. Three new themes emerged that had not appeared in first order constructs: the help-seeking journey; healthcare services' ability to address DVA; and under-reporting of relationship difficulties.

Help-seeking journey

Researchers described how participants accessed healthcare services through different routes: a reactive route, following contact with other services (often the police or social

For peer review only - http://bmjopen.bmj.com/site/about/guidelines.xhtml

services) due to their abusive behaviour, or a proactive route, when individuals sought help directly through healthcare for their relationship problems or a health problem related to their abusive behaviours. Researchers interpreted that individuals who sought help proactively and were explicit about their use of abusive behaviours found the help they desired more easily. Usually this was in the form of accessing a treatment programme for DVA perpetrators or another intervention directly addressing abusive behaviour[25].

Some individuals sought help for their relationship problems in indirect ways, by reporting anger[23,25] or common mental health problems such as depressive symptoms[25]. These participants reported finding it unhelpful when doctors responded to this type of help-seeking by prescribing antidepressant medication or referring service users to general counselling services without exploring the reasons behind their symptoms.

Some participants hoped to access specific support to reduce abusive behaviours, often framed as 'anger management services', and were frustrated when those services proved difficult to find or to access. Researchers interpreted that the ability to access services promptly was crucial, as individuals were often ambivalent towards behavioural change and might have changed their minds about help-seeking if there was a long wait to access a service[25].

Healthcare services' ability to address DVA

Researchers suggested that participants' willingness to discuss relationship difficulties with healthcare staff were affected by what they perceived to be limitations of healthcare services[23,24].

In the addictions setting, researchers identified that participants were aware of time constraints within services and decided to use their consultations to discuss problems other than their relationship difficulties. Professionals' lack of expertise to address relationship problems was also mentioned by addiction service users as a barrier to disclosure, though it was not clear to the researchers how that perception had developed, and whether it had originated in participants' beliefs about the appropriateness of discussing relationship problems in addictions services. It was noted by the researchers that many participants reported that they had never been asked about their relationships by clinicians in that setting[23].

Concerns about clinicians' lack of time or skills to address relationship difficulties were also identified by researchers in the study located in a sexual health clinic: participants suggested that the sexual health clinic was a more appropriate venue for inquiry and disclosure of relationship problems than primary care, which was perceived as a place

For peer review only - http://bmjopen.bmj.com/site/about/guidelines.xhtml

where staff did not have the skill or time to explore issues about domestic abuse. Sexual health clinics were also seen by participants in that study as a more 'private' setting where sensitive and intimate issues could be discussed. Researchers recommended training for staff in healthcare services to increase awareness of DVA and improve their communication skills to help build trusting relationships with service users[24].

Under-reporting of relationship difficulties

Some participants under-reported DVA perpetration, which researchers interpreted as a tendency to minimise the seriousness of DVA, shame associated with DVA and fear of the consequences of disclosure, as identified in the first order constructs. In two studies, participants denied having ever perpetrated abuse, despite having been convicted of DVA-related offences or having answered positively to questions about DVA perpetration in the quantitative survey in the same study[23,26]. Researchers concluded that the denial or lack of awareness indicated a mismatch between actions (even those that participants had admitted to) and perception of what constitutes DVA, which may also contribute to the tendency towards under-reporting of DVA perpetration to healthcare professionals[23,24] in addition to other factors such as shame and fear of the adverse consequences of disclosure.

Table 5. Second-order constructs

**Themes**	**Second order constructs**
Help-seeking journey	Reactive vs. proactive help-seeking<br><br>Implicit vs. explicit help-seeking
Healthcare services' ability to address DVA	Time constraints<br><br>Healthcare services' time constraints and competing priorities<br><br>IPV 'not a legitimate issue' to discuss with healthcare staff<br><br>Relevant expertise
Under-reporting of relationship difficulties	Minimisation of DVA

For peer review only - http://bmjopen.bmj.com/site/about/guidelines.xhtml

	Fear of consequences<br><br>Shame about DVA<br><br>Understanding about DVA

Third-order constructs:

The constructs identified in these studies were suited for the creation of a line or argument synthesis; there were no major or unexplained contradictions within studies or between studies. Additionally, the studies included in this review explored different aspects of healthcare experiences, which complemented one another. The third order constructs originated from this meta ethnography can be divided in factors that facilitate DVA perpetrators' disclosure of relationship difficulties to healthcare staff and their engagement with healthcare services; and factors that act as barriers to disclosure or DVA or healthcare service engagement (see Table 6).

Factors that facilitate DVA perpetrators' disclosure of DVA to healthcare staff and help-seeking

Some individuals reported having explicitly requested help to reduce abusive behaviours, in some cases triggered by a "crisis" (such as separation from a partner, escalation of abuse, or arrest), and sought or expected a specific intervention to address their problems. Others spoke about abuse, but used somewhat vague terms, referring to 'relationship problems', 'anger' or reporting low mood. Active listening by professionals, further exploration of relationship history, skills in enquiring about relationship difficulties sensitively and linking these to service users' health concerns tended to facilitate engagement of DVA perpetrators with healthcare services. The availability of on-site support for relationship problems may also act as a facilitator and may be a necessary condition for some to disclose their relationship problems. It was unclear what form this immediate support needs to take beyond non-judgmental listening by professionals or practical support.

Factors that act as barriers to disclosure of DVA and engagement with healthcare services

The studies demonstrated that perpetrators' perceptions that healthcare professionals lack expertise to address DVA and their own low prioritization of relationship problems negatively affect willingness to disclose relationship difficulties to healthcare staff.

For peer review only - http://bmjopen.bmj.com/site/about/guidelines.xhtml

Some individuals feel shame, guilt or regret when discussing abusive behaviours with healthcare staff, whereas others see abusive behaviours as a normal part of an intimate relationship. These factors affect whether (and how) individuals seek help. Concerns about being blamed for relationship problems and about confidentiality, particularly due to fears of involvement from other services (e.g., children's social services), also act as important barriers to disclosure of DVA to healthcare professionals. These concerns, combined with negative emotions and attitudes towards DVA, are some of the factors underlying the under-reporting of DVA by perpetrators.

Table 6. Third order constructs

Third order construct	Description
Facilitators of disclosure of DVA to healthcare staff and engagement with healthcare	Reaching a crisis point or experiencing negative social consequences following abusive behaviour<br><br>Active listening by healthcare professionals<br><br>Availability of emotional and practical support (ideally on-site)
Barriers to disclosure of DVA to healthcare staff and engagement with healthcare	Negative emotions and attitudes towards DVA by perpetrators<br><br>Fear of consequences of disclosure<br><br>Lack of trust in healthcare services' knowledge or expertise in addressing DVA

DISCUSSION

Main findings

Our review provides the first synthesis of qualitative research on the healthcare experiences of DVA perpetrators and identifies factors that influence the relationship of DVA perpetrators with healthcare professionals. Findings indicate that being listened to and being offered emotional and practical support facilitate disclosure of abusive behaviours to clinicians. Conversely, the barriers to the disclosure of abusive behaviours by perpetrators to healthcare workers include feelings of shame, beliefs that abuse is a normal

For peer review only - http://bmjopen.bmj.com/site/about/guidelines.xhtml

part of intimate relationships, concerns about confidentiality and a lack of trust in healthcare professionals' ability or readiness to address DVA.

The study also found that the negative consequences of DVA perpetration, such as involvement of children's social services, being banned from social spaces, or relationship breakups may act as barriers or facilitators to disclosure to healthcare services. This apparent contradiction can be explained by the context of the perpetrators' experiences and the timing of help-seeking. DVA perpetrators chose not to disclose their abusive behaviours to healthcare professionals when they wanted to avoid the negative social consequences of those behaviours, such as the perceived threat of losing contact with one's children via involvement of children's social services. In contrast, when perpetrators had already experienced the adverse consequences of abusive behaviours, such as relationship separation, escalation of abuse, or exclusion from social spaces, those events acted as triggers to disclosure and help-seeking. In these situations, perpetrators had decided to disclose their difficulties to clinicians to seek help and avoid further negative consequences. Importantly, some of these factors, such as relationship breakdown and escalation of abuse, are known to be risk factors for domestic homicides[28,29], and knowledge of those factors is crucial for the assessment of risk by healthcare professionals.

An intra-study contradiction was identified involving the constructs of attitudes and emotions related to DVA. Some participants expressed shame and regret over their behaviours, while others minimised or normalised DVA[23]. The minimization and normalisation are consistent with the findings of a recent qualitative study of male IPV perpetrators in treatment for addiction and their female partners, which examined the perspective of perpetrators and their partners on the role of substance use in IPV[30]. In that study, perpetrators tended to attribute IPV to isolated events triggered by specific disputes, while their partners described a pattern characterized by enduring abuse, including severe violence and coercive control in some cases. The intra-study contradiction identified in our review highlights how individual emotional reactions to DVA by perpetrators can vary, with emotions ranging from denial and normalisation to self-reflection, shame and regret over their actions.

Implications for clinical practice and policy

Our findings demonstrate some of the complexities associated with addressing DVA perpetration in healthcare services. Relationship difficulties and DVA can elicit a variety of reactions in perpetrators, some of which may be conflicting, and which may affect the likelihood of disclosure of abusive behaviours to healthcare professionals. Additionally, individuals may present to healthcare services with the aim to seek help for the consequences of relationship difficulties (e.g., low mood) without disclosing DVA

For peer review only - http://bmjopen.bmj.com/site/about/guidelines.xhtml

perpetration directly. This highlights the importance of trusting rapports between healthcare professionals and service users, as well as a willingness on the part of clinicians to explore the intimate and family relationships of their service users in more detail.

Some of the participants of the studies included in this review articulated that DVA was not a legitimate issue to bring to consultations with healthcare professionals[23], while others stated that it seemed appropriate to discuss DVA with clinicians in services that were perceived as more 'private', such as sexual health services[24]. Moreover, some participants were not aware of what behaviours constitute abuse[23,24]. This theme suggests that, beyond raising awareness of DVA perpetration among healthcare professionals, it may be necessary to raise public awareness of what behaviours constitute perpetration of abuse. Public awareness campaigns on DVA, including the campaign by the UK Home Office during the COVID-19 pandemic[31], often address survivors or those at risk of experiencing DVA; campaigns directed at perpetrators will likely need a different language and will need to be circulated through different channels to address the populations at risk of perpetrating DVA. For those campaigns to be effective, the training of health professionals (and others who are likely to encounter DVA, such as the police and social care) on how to safely enquire about DVA perpetration and how to respond to disclosures will be essential.

A number of participants reported that they expected to receive support from healthcare services to access interventions to reduce their anger or violence ('anger management'), or a treatment programme for DVA perpetrators. Although a systematic review of qualitative studies has demonstrated that users of IPV perpetrator programmes find value in those interventions[32], the evidence for effectiveness of DVA perpetrator programmes is limited[33-35]; most intervention studies are restricted to specific populations or settings, making them less generalizable, and many lack a control group. However, psychological therapies such as behavioural couples' therapy (BCT) or cognitive behavioural therapy (CBT) combined with treatment for alcohol misuse can be effective in reducing abusive behaviours[35-38]. This suggests that the existing role of healthcare services in identifying and treating substance use may provide a route into addressing DVA perpetration when those issues co-exist.

Strengths and limitations of this review

The number of studies found was small; twice as many studies were included in a meta-synthesis of the experiences of DVA survivors[39], indicating that DVA perpetration continues to be an under-researched topic relative to experiences of DVA. The healthcare experiences of DVA perpetrators were not the primary focus of included studies and made

For peer review only - http://bmjopen.bmj.com/site/about/guidelines.xhtml

up only a small proportion of the data presented. Therefore, a small number of first- and second-order constructs identified by each study were appropriate for synthesis.

This review did not identify any studies from non-Western, non-English speaking or low- and middle-income countries that met inclusion criteria, nor studies focusing on perpetrators of non-partner adult family violence or female perpetrators of DVA. This raises the question of whether the findings are generalizable to those populations.

CONCLUSION

Our results provide the first synthesis of evidence on the healthcare experiences of DVA perpetrators. Our meta-synthesis indicates that healthcare professionals need to be open to asking questions about service users' family and intimate relationships and be attentive to requests for help that may not be explicit. These findings can inform the development of training to improve the identification of DVA perpetration in healthcare settings. Our findings also suggest a possible role for public health campaigns directed at groups at risk of DVA perpetration to improve awareness of what constitutes abuse and encourage individuals to disclose those behaviours to healthcare professionals. DVA perpetration is a complex and multi-factorial that requires the involvement of a number of public and third sector organisations, and further research is needed in how best to integrate the responses of healthcare with those of other sectors, such as social care, police and criminal justice system.

Funding

Sponsors/funding: Dr Calcia is a self-funded MD(Res) student and received salary support through a 3-month full time secondment to the Section of Women's Mental Health, Health Service and Population Research Department, IoPPN, KCL, from July-September 2018, from an NIHR Research Professorship to Louise Howard (NIHR-RP-R3-12-011). Louise Howard is an NIHR Senior investigator. The funding organisations had no involvement in the study design; in the collection, analysis and interpretation of the data; in the writing of the report and in the decision to submit the study for publication.

Figure legend:

For peer review only - http://bmjopen.bmj.com/site/about/guidelines.xhtml

Figure 1: PRISMA Diagram

References

1. ONS, 2018 Office for National Statistics (2017, 25 May). Crime Survey for England and Wales (CSEW). Domestic abuse, sexual assault and stalking. Retrieved from: https://www.ons.gov.uk/peoplepopulationandcommunity/crimeandjustice/compendium/focusonviolentcrimeandsexualoffences/yearendingmarch2016/domesticabusesexualassaultandstalking

2. UK Home Office. Domestic Abuse Bill 2020: overarching documents. 3 March 2020. [cited July 2020] .Available from: https://www.gov.uk/government/publications/domestic-abuse-bill-2020-overarching-documents

3. Campbell JC. Health consequences of intimate partner violence. The lancet. 2002 Apr 13;359(9314):1331-6.

4. Trevillion K, Oram S, Feder G, et al. Experiences of domestic violence and mental disorders: a systematic review and meta-analysis. PloS one. 2012 Dec 26;7(12):e51740.

5. Howard LM, Oram S, Galley H, et al. Domestic violence and perinatal mental disorders: a systematic review and meta-analysis. PLoS Med. 2013 May 28;10(5):e1001452.

6. Edleson JL. Children's witnessing of adult domestic violence. Journal of interpersonal Violence. 1999 Aug;14(8):839-70.

7. Kitzmann KM, Gaylord NK, Holt AR, Kenny ED. Child witnesses to domestic violence: a meta-analytic review. Journal of consulting and clinical psychology. 2003 Apr;71(2):339.

8. Flach C, Leese M, Heron J, Evans J, Feder G, Sharp D, Howard LM. Antenatal domestic violence, maternal mental health and subsequent child behaviour: a cohort study. BJOG: An International Journal of Obstetrics & Gynaecology. 2011 Oct;118(11):1383-91.

For peer review only - http://bmjopen.bmj.com/site/about/guidelines.xhtml

9. Oram S, Trevillion K, Feder G, Howard LM. Prevalence of experiences of domestic violence among psychiatric patients: systematic review. The British Journal of Psychiatry. 2013 Feb;202(2):94- https://doi.org/10.1192/bjp.bp.112.109934

10. Yu R, Nevado-Holgado AJ, Molero Y, D'Onofrio BM, Larsson H, Howard LM, Fazel S. Mental disorders and intimate partner violence perpetrated by men towards women: A Swedish population-based longitudinal study. PLoS medicine. 2019 Dec 17;16(12):e1002995. https://doi.org/10.1371/journal.pmed.1002995

11. Khalifeh H, Johnson S, Howard LM, Borschmann R, Osborn D, Dean K, Hart C, Hogg J, Moran P. Violent and non-violent crime against adults with severe mental illness. The British Journal of Psychiatry. 2015 Apr;206(4):275-82. https://doi.org/10.1192/bjp.bp.114.147843

12. Hester M, Ferrari G, Jones SK, Williamson E, Bacchus LJ, Peters TJ, Feder G. Occurrence and impact of negative behaviour, including domestic violence and abuse, in men attending UK primary care health clinics: a cross-sectional survey. BMJ open. 2015 May 1;5(5). http://dx.doi.org/10.1136/bmjopen-2014-007141

13. Moher D, Liberati A, Tetzlaff J, Altman DG, The PRISMA Group. Preferred Reporting Items for Systematic Reviews and Meta-Analyses: The PRISMA Statement

14. Holdsworth E, Bowen E, Brown S, Howat D. Offender engagement in group programs and associations with offender characteristics and treatment factors: A review. Aggression and Violent Behavior. 2014 Mar 1;19(2):102-21. https://doi.org/10.1016/j.avb.2014.01.004

15. Noblit GW, Hare RD. Meta-ethnography: Synthesizing qualitative studies. sage; 1988.

16. Pilkington H. Employing meta-ethnography in the analysis of qualitative data sets on youth activism: a new tool for transnational research projects?. Qualitative Research. 2018 Feb;18(1):108-30. https://doi.org/10.1177/1468794117707805

17. Viswanathan M, Patnode CD, Berkman ND, et al. Assessing the risk of bias in systematic reviews of health care interventions. InMethods guide for effectiveness and comparative effectiveness reviews [Internet] 2017 Dec 13. Agency for Healthcare Research and Quality (US). https://doi.org/10.23970/AHRQEPCMETHGUIDE2.

For peer review only - http://bmjopen.bmj.com/site/about/guidelines.xhtml

18. Harden A, Brunton G, Fletcher A, et al. Teenage pregnancy and social disadvantage: systematic review integrating controlled trials and qualitative studies. Bmj. 2009 Nov 13;339:b4254. https://doi.org/10.1136/bmj.b4254

19. Daly J, Willis K, Small R, et al. A hierarchy of evidence for assessing qualitative health research. Journal of clinical epidemiology. 2007 Jan 1;60(1):43-9. https://doi.org/10.1016/j.jclinepi.2006.03.014

20. Dixon-Woods M, Shaw RL, Agarwal S, et al. The problem of appraising qualitative research. BMJ Quality & Safety. 2004 Jun 1;13(3):223-5. http://dx.doi.org/10.1136/qshc.2003.008714

21. van Rooij FB, Ten Haaf J, Verhoeff AP. Temporary restraining orders in the Netherlands: a qualitative examination of perpetrator and victim views. Journal of Family Violence. 2013 Jul 1;28(5):503-14. https://doi.org/10.1007/s10896-013-9520-2

22. Morgan K, Williamson E, Hester M, et al. Asking men about domestic violence and abuse in a family medicine context: Help seeking and views on the general practitioner role. Aggression and violent behavior. 2014 Nov 1;19(6):637-42. https://doi.org/10.1016/j.avb.2014.09.008

23. Hashimoto N, Radcliffe P, Gilchrist G. Help-seeking behaviors for intimate partner violence perpetration by men receiving substance use treatment: a mixed-methods secondary analysis. Journal of interpersonal violence. 2018 May 1:0886260518770645. https://doi.org/10.1177/0886260518770645

24. Bacchus LJ, Buller AM, Ferrari G, et al. "It's Always Good to Ask": a mixed methods study on the perceived role of sexual health practitioners asking gay and bisexual men about experiences of domestic violence and abuse. Journal of Mixed Methods Research. 2018 Apr;12(2):221-43. https://doi.org/10.1177/1558689816651808

25. Hester M, Westmarland N, Gangoli G, et al. Domestic violence perpetrators: identifying needs to inform early intervention. Newcastle: Northern Rock Foundation. 2006 Apr

26. Swogger R. Incarcerated Men and the Etiology of Intimate Partner Violence [PhD thesis]. Antioch University. 2016. http://aura.antioch.edu/cgi/viewcontent.cgi?article=1309&context=etds

For peer review only - http://bmjopen.bmj.com/site/about/guidelines.xhtml

27. Corbin JM, Strauss A. Grounded theory research: Procedures, canons, and evaluative criteria. Qualitative sociology. 1990 Mar 1;13(1):3-21. https://doi.org/10.1007/BF00988593

28. Dawson M, Piscitelli A. Risk factors in domestic homicides: Identifying common clusters in the Canadian context. Journal of interpersonal violence. 2017 Sep 1:0886260517729404. https://doi.org/10.1177%2F0886260517729404

29. UK Home Office. Domestic Homicide Reviews: key findings from analysis of domestic homicide reviews. December 2016 [cited Jul 2020]. Available from: https://assets.publishing.service.gov.uk/government/uploads/system/uploads/attachment_data/file/575232/HO-Domestic-Homicide-Review-Analysis-161206.pdf

30. Radcliffe P, Gadd D, Henderson J, et al. What role does substance use play in intimate partner violence? A narrative analysis of in-depth interviews with men in substance use treatment and their current or former female partner. Journal of interpersonal violence. 2019 Oct 3:0886260519879259. https://doi.org/10.1177%2F0886260519879259

31. UK Home Office. Home Secretary's Statement on domestic abuse and coronavirus (COVID-19): 11 April 2020 [cited Jul 2020]. Available from: https://www.gov.uk/government/speeches/home-secretary-outlines-support-for-domestic-abuse-victims

32. McGinn T, McColgan M, Taylor B. Male IPV perpetrator's perspectives on intervention and change: A systematic synthesis of qualitative studies. Trauma, Violence, & Abuse. 2020 Jan;21(1):97-112. https://doi.org/10.1177%2F1524838017742167

33. Eckhardt CI, Murphy CM, Whitaker DJ, et al. The effectiveness of intervention programs for perpetrators and victims of intimate partner violence. Partner abuse. 2013 Jan 1;4(2):196-231. https://psycnet.apa.org/doi/10.1891/1946-6560.4.2.196

34. National Institute for Health and Care Excellence. Domestic violence and abuse: multi-agency working. NICE; 2014. (Public Health guideline [PH50]). Available from: https://www.nice.org.uk/guidance/ph50

For peer review only - http://bmjopen.bmj.com/site/about/guidelines.xhtml

35. Tarzia L, Forsdike K, Feder G, et al. Interventions in health settings for male perpetrators or victims of intimate partner violence. Trauma, Violence, & Abuse. 2020 Jan;21(1):123-37. https://doi.org/10.1177%2F1524838017744772

36. Fals-Stewart W, Clinton-Sherrod M. Treating intimate partner violence among substance-abusing dyads: The effect of couples therapy. Professional Psychology: Research and Practice. 2009 Jun;40(3):257. https://psycnet.apa.org/doi/10.1037/a0012708

37. O'Farrell TJ, Murphy CM, Stephan SH, et al. Partner violence before and after couples-based alcoholism treatment for male alcoholic patients: the role of treatment involvement and abstinence. Journal of consulting and clinical psychology. 2004 Apr;72(2):202. https://psycnet.apa.org/doi/10.1037/0022-006X.72.2.202

38. Satyanarayana VA, Nattala P, Selvam S, et al. Integrated cognitive behavioral intervention reduces intimate partner violence among alcohol dependent men, and improves mental health outcomes in their spouses: A clinic based randomized controlled trial from South India. Journal of substance abuse treatment. 2016 May 1;64:29-34. https://doi.org/10.1016/j.jsat.2016.02.005

39. Trevillion K, Hughes B, Feder G, et al. Disclosure of domestic violence in mental health settings: A qualitative meta-synthesis. International Review of Psychiatry. 2014 Aug 1;26(4):430-44. https://doi.org/10.3109/09540261.2014.924095

For peer review only

Figure 1: PRISMA Diagram

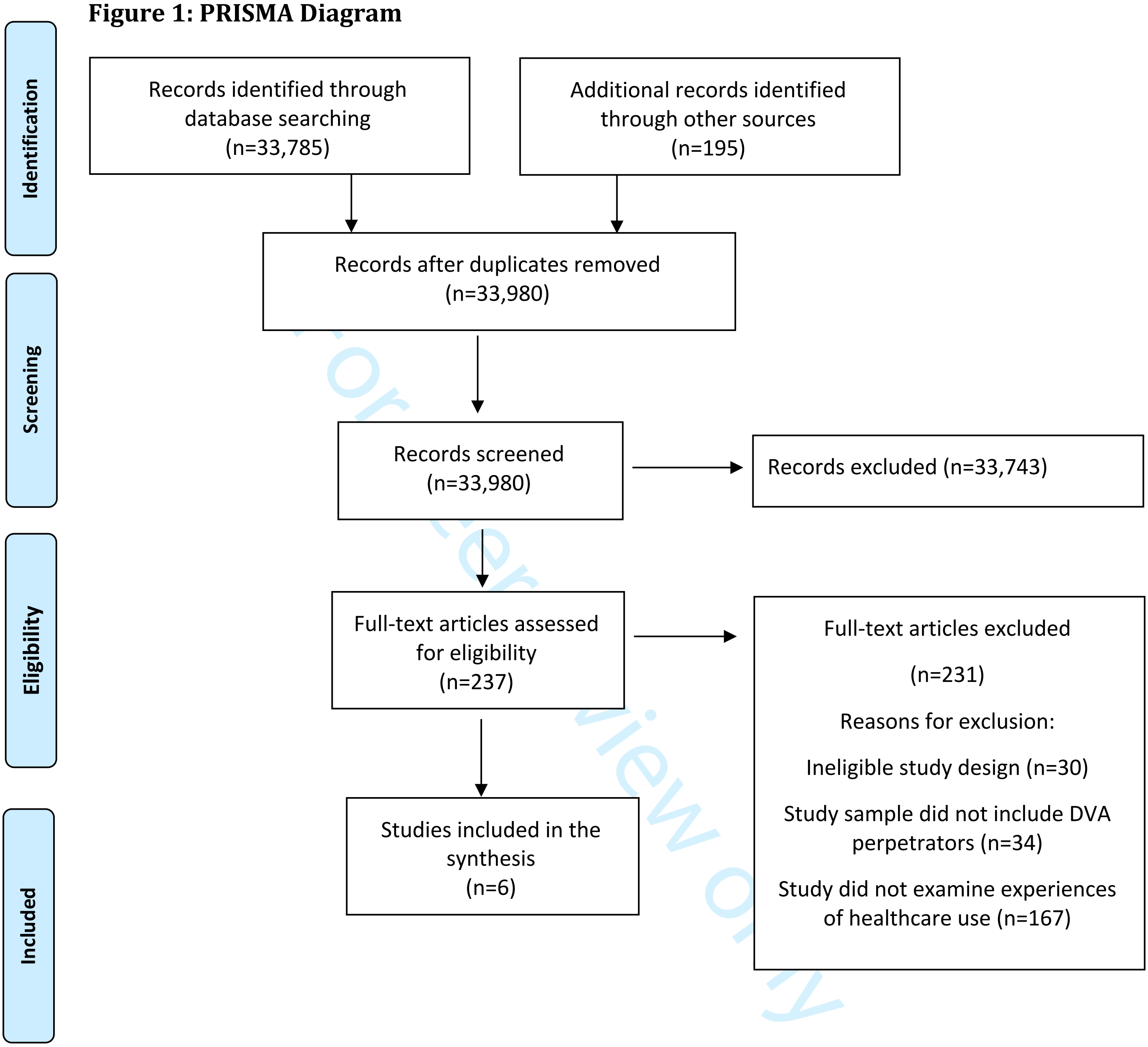

For peer review only - http://bmjopen.bmj.com/site/about/guidelines.xhtml

Reporting checklist for systematic review and meta-analysis.

Based on the PRISMA guidelines.

Instructions to authors

Complete this checklist by entering the page numbers from your manuscript where readers will find each of the items listed below.

Your article may not currently address all the items on the checklist. Please modify your text to include the missing information. If you are certain that an item does not apply, please write "n/a" and provide a short explanation.

Upload your completed checklist as an extra file when you submit to a journal.

In your methods section, say that you used the PRISMAreporting guidelines, and cite them as:

Moher D, Liberati A, Tetzlaff J, Altman DG, The PRISMA Group. Preferred Reporting Items for Systematic Reviews and Meta-Analyses: The PRISMA Statement

		Reporting Item	Page Number
**Title**			
	#1	Identify the report as a systematic review, meta-analysis, or both.	1, 2
**Abstract**			
Structured summary	#2	Provide a structured summary including, as applicable: background; objectives; data sources; study eligibility criteria, participants, and interventions; study appraisal and synthesis methods; results; limitations; conclusions and implications of key findings; systematic review registration number	2
**Introduction**			
Rationale	#3	Describe the rationale for the review in the context of what is already known.	3

For peer review only

Objectives	#4	Provide an explicit statement of questions being addressed with reference to participants, interventions, comparisons, outcomes, and study design (PICOS).	4, 5
**Methods**			
Protocol and registration	#5	Indicate if a review protocol exists, if and where it can be accessed (e.g., Web address) and, if available, provide registration information including the registration number.	1
Eligibility criteria	#6	Specify study characteristics (e.g., PICOS, length of follow-up) and report characteristics (e.g., years considered, language, publication status) used as criteria for eligibility, giving rational	4, 5
Information sources	#7	Describe all information sources in the search (e.g., databases with dates of coverage, contact with study authors to identify additional studies) and date last searched.	5, 6
Search	#8	Present full electronic search strategy for at least one database, including any limits used, such that it could be repeated.	6
Study selection	#9	State the process for selecting studies (i.e., for screening, for determining eligibility, for inclusion in the systematic review, and, if applicable, for inclusion in the meta-analysis).	6, 7
Data collection process	#10	Describe the method of data extraction from reports (e.g., piloted forms, independently by two reviewers) and any processes for obtaining and confirming data from investigators.	6, 7
Data items	#11	List and define all variables for which data were sought (e.g., PICOS, funding sources), and any assumptions and simplifications made.	7
Risk of bias in individual studies	#12	Describe methods used for assessing risk of bias in individual studies (including specification of whether this was done at the study or outcome level, or both), and how this information is to be used in any data synthesis.	7, 8, 9
Summary measures	#13	State the principal summary measures (e.g., risk ratio, difference in means).	n/a (the study is a meta synthesis of qualitative studies)

For peer review only

Planned methods of analyis	#14	Describe the methods of handling data and combining results of studies, if done, including measures of consistency (e.g., I2) for each meta-analysis.	7
Risk of bias across studies	#15	Specify any assessment of risk of bias that may affect the cumulative evidence (e.g., publication bias, selective reporting within studies).	7, 8, 9
Additional analyses	#16	Describe methods of additional analyses (e.g., sensitivity or subgroup analyses, meta-regression), if done, indicating which were pre-specified.	n/a (the study is a meta synthesis of qualitative studies)
**Results**			
Study selection	#17	Give numbers of studies screened, assessed for eligibility, and included in the review, with reasons for exclusions at each stage, ideally with a flow diagram.	9, 10
Study characteristics	#18	For each study, present characteristics for which data were extracted (e.g., study size, PICOS, follow-up period) and provide the citation.	10-17
Risk of bias within studies	#19	Present data on risk of bias of each study and, if available, any outcome-level assessment (see Item 12).	10, 11
Results of individual studies	#20	For all outcomes considered (benefits and harms), present, for each study: (a) simple summary data for each intervention group and (b) effect estimates and confidence intervals, ideally with a forest plot.	n/a (the study is a meta synthesis of qualitative studies)
Synthesis of results	#21	Present the main results of the review. If meta-analyses are done, include for each, confidence intervals and measures of consistency.	17-19
Risk of bias across studies	#22	Present results of any assessment of risk of bias across studies (see Item 15).	n/a
Additional analysis	#23	Give results of additional analyses, if done (e.g., sensitivity or subgroup analyses, meta-regression [see Item 16]).	n/a (the study is a meta synthesis of qualitative studies)

Discussion

For peer review only

Summary of Evidence	#24	Summarize the main findings, including the strength of evidence for each main outcome; consider their relevance to key groups (e.g., health care providers, users, and policy makers	19-21
Limitations	#25	Discuss limitations at study and outcome level (e.g., risk of bias), and at review level (e.g., incomplete retrieval of identified research, reporting bias).	21
Conclusions	#26	Provide a general interpretation of the results in the context of other evidence, and implications for future research.	21, 22
**Funding**			
Funding	#27	Describe sources of funding or other support (e.g., supply of data) for the systematic review; role of funders for the systematic review.	1

Notes:

- 13: n/a (the study is a meta synthesis of qualitative studies)
- 16: n/a (the study is a meta synthesis of qualitative studies)
- 20: n/a (the study is a meta synthesis of qualitative studies)
- 23: n/a (the study is a meta synthesis of qualitative studies) The PRISMA checklist is distributed under the terms of the Creative Commons Attribution License CC-BY. This checklist was completed on 24. July 2020 using https://www.goodreports.org/, a tool made by the EQUATOR Network in collaboration with Penelope.ai

For peer review only

BMJ Open

The healthcare experiences of perpetrators of domestic violence and abuse: a systematic review and meta-synthesis

Journal:	*BMJ Open*
Manuscript ID	bmjopen-2020-043183.R1
Article Type:	Original research
Date Submitted by the Author:	06-Apr-2021
Complete List of Authors:	Calcia, Marilia; King's College London, Section of Women's Mental Health, Department of Health Service and Population Research, Institute of Psychiatry, Psychology and Neuroscience<br>Bedi, Simran; King's College London, Section of Women's Mental Health, Department of Health Service and Population Research, Institue of Psychiatry, Psychology and Neuroscience<br>Howard, Louise; King's College London, Section of Women's Mental Health, Department of Health Service and Population Research, Institute of Psychiatry, Psychology and Neuroscience<br>Lempp, Heidi; King's College London, Centre for Rheumatic Diseases, Department of Inflammation Biology, Faculty of Life Sciences and Medicine<br>Oram, Sian; King's College London, Section of Women's Mental Health, Department of Health Service and Population Research, Institute of Psychiatry, Psychology and Neuroscience
**Primary Subject Heading**:	Health services research
Secondary Subject Heading:	Qualitative research
Keywords:	MENTAL HEALTH, PRIMARY CARE, Substance misuse < PSYCHIATRY, Forensic psychiatry < PSYCHIATRY, QUALITATIVE RESEARCH, SEXUAL MEDICINE

SCHOLARONE™
Manuscripts

I, the Submitting Author has the right to grant and does grant on behalf of all authors of the Work (as defined in the below author licence), an exclusive licence and/or a non-exclusive licence for contributions from authors who are: i) UK Crown employees; ii) where BMJ has agreed a CC-BY licence shall apply, and/or iii) in accordance with the terms applicable for US Federal Government officers or employees acting as part of their official duties; on a worldwide, perpetual, irrevocable, royalty-free basis to BMJ Publishing Group Ltd ("BMJ") its licensees and where the relevant Journal is co-owned by BMJ to the co-owners of the Journal, to publish the Work in this journal and any other BMJ products and to exploit all rights, as set out in our licence.

The Submitting Author accepts and understands that any supply made under these terms is made by BMJ to the Submitting Author unless you are acting as an employee on behalf of your employer or a postgraduate student of an affiliated institution which is paying any applicable article publishing charge ("APC") for Open Access articles. Where the Submitting Author wishes to make the Work available on an Open Access basis (and intends to pay the relevant APC), the terms of reuse of such Open Access shall be governed by a Creative Commons licence – details of these licences and which Creative Commons licence will apply to this Work are set out in our licence referred to above.

Other than as permitted in any relevant BMJ Author's Self Archiving Policies, I confirm this Work has not been accepted for publication elsewhere, is not being considered for publication elsewhere and does not duplicate material already published. I confirm all authors consent to publication of this Work and authorise the granting of this licence.

For peer review only

The healthcare experiences of perpetrators of domestic violence and abuse: a systematic review and meta-synthesis

Marilia A Calcia, Simran Bedi, Louise M Howard, Heidi Lempp, Sian Oram

PROSPERO registration id: CRD42017073818

Authors, affiliation and contact:

Dr Marilia A. Calcia, Doctoral student, Health Service and Population Research Department, Institute of Psychiatry, Psychology and Neuroscience (IoPPN), King's College London (KCL) marilia.a.calcia@kcl.ac.uk
Simran Bedi, MBBS Student, KCL (Simran.bedi@kcl.ac.uk)
Prof Louise M Howard, Professor of Women's Mental Health, Health Service and Population Research Department, IoPPN, KCL (louise.howard@kcl.ac.uk)
Dr Heidi Lempp, Reader in Medical Sociology, Centre for Rheumatic Diseases, Department of Inflammation Biology, Faculty of Life Sciences and Medicine, KCL (heidi.lempp@kcl.ac.uk)
Dr Sian Oram, Senior Lecturer in Women's Mental Health and Head of the Section of Women's Mental Health, Health Service and Population Research Department, IoPPN, KCL (sian.oram@kcl.ac.uk)

Corresponding author:

Dr Marilia A. Calcia (marilia.a.calcia@kcl.ac.uk)

Abstract

Objectives: Domestic violence and abuse (DVA) is highly prevalent, with severe adverse consequences to the health and wellbeing of survivors. There is a smaller evidence base on the health of DVA perpetrators and their engagement with healthcare services. This review examines the experiences of perpetrators of DVA of accessing healthcare services and the barriers and facilitators to their disclosure of abusive behaviours in these settings.

Design: A systematic review and meta-synthesis of qualitative studies.

For peer review only
For peer review only - http://bmjopen.bmj.com/site/about/guidelines.xhtml

Data sources: Qualitative studies published from the start of each database until May 2018, were identified in Cochrane, MEDLINE, EMBASE, PsycINFO, HMIC, BNID, CINAHL, ASSIA, IBSS, SSCI (peer-reviewed literature) and NDLTD, Open Grey and SCIE Online (grey literature). The databases were searched from their respective start dates to 15 March 2020. Eligibility criteria required studies used qualitative or mixed methods to report on the experiences of healthcare use by perpetrators of DVA. A meta-ethnographic method was used to analyse the extracted data.

Results: Of 33,785 papers identified, 6 studies (n=125 participants; 124 men, 1 woman) met the inclusion criteria. Barriers to disclosure of DVA to healthcare staff included perpetrators' negative emotions and attitudes towards their abusive behaviours; fear of consequences of disclosure; and lack of trust in healthcare services' ability to address DVA. Facilitators of disclosure of DVA and engagement with healthcare services were experiencing social consequences of abusive behaviours; feeling listened to by healthcare professionals; and offers of emotional and practical support for relationship problems by healthcare staff.

Conclusions: DVA perpetration is a complex issue with multiple barriers to healthcare engagement and disclosure. However, healthcare services can create positive conditions for the engagement of individuals who perpetrate abuse.

Article summary:

Strengths and limitations of this study:

- This study provides a systematic review of the healthcare experiences of domestic violence and abuse (DVA) perpetrators, including facilitators and barriers to disclosure of abusive behaviours to healthcare professionals
- Robust procedures for systematic reviewing and quality assessment were adopted, in line with Preferred Reporting Items for Systematic Reviews and Meta-Analyses reporting guidelines.
- The number of studies identified was small and some healthcare settings, e.g., emergency department, were not represented in the study sample.
- This review identified studies conducted in high-income countries and most participants were men who perpetrated intimate partner violence (IPV); the findings may, therefore, be less applicable to other settings such as low- and middle-income countries, or other populations such as perpetrators of family violence (FV) or female perpetrators of DVA.

For peer review only - http://bmjopen.bmj.com/site/about/guidelines.xhtml

For peer review only

Keywords: mental health; primary care; substance misuse; forensic psychiatry; sexual medicine; qualitative research

Word count: 7,304

INTRODUCTION

Domestic violence and abuse (DVA) are highly prevalent worldwide. According to the World Health Organisation (WHO), 27% of women aged 15-49 years who have been in an intimate relationship have experienced physical or sexual intimate partner violence (IPV) and 38% of female victims of homicide have been killed by an intimate partner[1]. In the UK, the definition of domestic violence includes family violence as well as IPV. DVA is defined in the UK Domestic Abuse Bill 2020 as abusive behaviour (which may be physically, sexually, emotionally, psychologically or economically abusive or violent, threatening, controlling or coercive) by one person to another, when both are aged 16 or over and are "personally connected". The term personally connected refers to two people who are, or have been, in an intimate personal relationship, marriage or civil partnership, where each has parental responsibility to a child, or who are relatives[2]. In the year ending March 2018, an estimated two million people experienced domestic violence and abuse (DVA) in England and Wales[3]. Female victims outnumbered male victims approximately two to one.

DVA has a range of impacts on survivors' health; up to 42% of women affected by IPV have reported injuries as a consequence of it[4]. Chronic pain, gastrointestinal problems, gynaecological problems, depression, anxiety, and other mental disorders are also known to be associated with DVA[5-7]. Women who have experienced IPV are at higher risk of having a low-birth-weight baby, abortions, depression and of acquiring HIV than women with no history of IPV ([4]). The adverse outcomes of DVA also affect children; children in families where DVA occurs have an increased risk of adverse behavioural, emotional and cognitive outcomes[8-10].

Less evidence exists regarding the health of DVA perpetrators, of which most relates to mental health. Systematic review evidence suggested both men and women with mental disorders are more likely to have a lifetime history of physical abuse towards a partner than those without a mental disorder, and the risk is higher in men than women[11]. A recent longitudinal study indicated that comorbid diagnoses of substance use and personality disorders increase the risk of intimate partner violence perpetration against women in men with mental disorders[12]. However, few studies reported on recent violence (within the past year), and there was little data on whether violence occurred during acute episodes of mental illness or on the role of substance use as a confounder.

For peer review only - http://bmjopen.bmj.com/site/about/guidelines.xhtml

Evidence regarding identification of DVA perpetration in both mental health and other healthcare settings is lacking. Qualitative studies have indicated that mental health professionals have little confidence in enquiring about and responding to DVA in general, and specifically about DVA perpetration[13 14] . A medical records review of male patients in UK primary care showed that only 0.5% had DVA perpetration or victimisation documented in their medical records, despite 16% of the same patients disclosing negative behaviours in their relationship with a partner in a contemporaneous survey [15]. A systematic review has highlighted that mental health service users are in favour of routine enquiry of experiences of DVA by healthcare professionals and value when clinicians respond to disclosure by being non-discriminatory and supportive towards those who reported having experienced DVA[6]. There is a small but emerging literature exploring the experiences of DVA perpetrators through qualitative studies, but to our knowledge no review has sought to synthesise evidence on DVA perpetrators' experiences of engaging with healthcare services or of the facilitators and barriers to disclosure of DVA perpetration to healthcare professionals.

This review aims to add to the evidence base by systematically identifying and reviewing qualitative studies that have explored the experiences of DVA perpetrators who access healthcare services, and by using meta-ethnography to synthesise and produce a new interpretation of this phenomenon. The review aimed to address the following questions:

(1) What are DVA perpetrators' experiences of being asked about and discussing DVA with healthcare staff?
(2) What are DVA perpetrators' experiences of using healthcare services when their history of DVA perpetration is known to healthcare staff?
(3) What are DVA perpetrators' experiences of seeking, accessing or being referred to for support to address their relationship difficulties and abusive behaviours by healthcare staff?
(4) What are DVA perpetrators' views on the association between their health and their relationship difficulties and abusive behaviours?

METHODS

We used the PRISMA reporting checklist when writing our report[16] and the eMERGE checklist for reporting of meta-ethnographies[17]. Please see Figure 1 for PRISMA checklist and Appendix 1 for eMERGE checklist.

Inclusion criteria

For peer review only - http://bmjopen.bmj.com/site/about/guidelines.xhtml

Studies meeting the following criteria were included:

- Population: Adults (> 16 years of age) of any gender who are known to be, or identified themselves as, current or former perpetrators of DVA. DVA is defined as any incident, or pattern of incidents, of controlling, coercive or threatening behaviour, violence or abuse, between individuals 16 years or older who are, or have been, intimate partners or family members, regardless of gender or sexuality.
- Phenomenon of interest: Experiences of using healthcare services
- Setting: Healthcare services (primary care, emergency medicine, mental health services, secondary care medical or surgical specialties)
- Design: Any qualitative or mixed methods study design
- Type: Studies published in peer-reviewed journals, in the grey literature (ineligible formats listed under exclusion criteria), including masters' dissertations and doctoral theses
- Language of publication: English, Spanish, Portuguese or Italian
- Date of publication: Published from the start date of each database until 15 March 2020

Exclusion criteria:

- Design: Studies not using qualitative methods
- Setting: Studies where the data reported experiences of use of treatment programmes for DVA perpetrators ('perpetrator programmes'). Perpetrator programmes are interventions with specific aims that do not always involve healthcare services and have been studied separately from the wider healthcare literature[18]
- Type: Book chapters, conference papers, editorials, letters or general comment papers
- Language of publication: Studies published in languages other than English, Spanish, Italian or Portuguese
- Studies where the published data were not sufficiently detailed to allow inclusion in the meta-synthesis, such as when participant accounts and/or researchers' interpretations were not clearly stated, or when data collection and analysis methods were not reported.

No geographic restrictions were set for this review.

Information sources

For peer review only - http://bmjopen.bmj.com/site/about/guidelines.xhtml

The following electronic bibliographic databases were searched: Cochrane, MEDLINE, EMBASE, PsycINFO, HMIC, BNID, CINAHL, ASSIA, IBSS, SSCI for peer-reviewed literature and NDLTD, OpenGrey and SCIE Online for grey literature.

Additionally, the primary reviewer conducted forwards citation tracking of included articles using Web of Science and Google Search.

Search strategy

The search strategy used combinations of search words for DVA, IPV or family violence; for qualitative research; for perpetration of violence and abuse; and for healthcare settings, to ensure that the search results matched the study questions and inclusion criteria. Please see Table 1 for search terms. The databases were searched from their respective start dates to 15 March 2020.

For peer review only

Table 1. Search terms

**DVA act - OR**	**Qualitative Research - OR**	**DVA perpetrator - OR**
Intimate partner violence;<br>Intimate partner abuse; Partner abuse OR violence<br>Spouse abuse OR violence;<br>Battered wom*;<br>Couple* abuse OR violence;<br>Domestic abuse OR violence;<br>Family abuse OR violence;<br>Coercive control;<br>Control$ behavio$r;<br>((abus$ OR batter$ OR violen$ OR beat$) adj2 (domestic OR partner$ OR family OR families OR spouse OR woman OR women OR men OR man OR female$ OR male$ OR wife OR wives OR husband$ OR boyfriend$ OR girlfriend$ OR elder$ OR brother$ OR sister$ OR father$ OR mother$ OR daughter$ OR son$)<br>(domestic adj2 homicid$).mp	Qualitative research/<br>Interview/<br>Theme$ or thematic<br>Qualitative study;<br>Qualitative<br>Ethnograph$;<br>Narrative;<br>Account;<br>Participant observation;<br>Grounded theory;<br>Interpretative;<br>Phenomenological Analysis;<br>Case stud$;<br>Focus group;<br>Framework analysis;<br>Framework approach;<br>Mixed-method$<br>Content analysis<br>Discourse analysis	Perpetrator$; Offender$;<br>Batterer$; Abuser$<br><br>**Setting:**<br>Patient$ OR<br>Service user$ OR<br>Consumer of (mental)health care<br>Health$ OR<br>Healthcare<br>Service$<br>Clinic$<br>Hospital$<br>Emergenc$<br>Outpatient

For peer review only - http://bmjopen.bmj.com/site/about/guidelines.xhtml

Patient and public involvement statement:

Patients or the public were not involved in the design, or conduct, or reporting, or dissemination plans of our research.

STUDY RECORDS

Data management:

Records were managed using EndNote software. The studies were coded using NVivo 11 software[[19]] and data were extracted into MS Excel.

Selection process:

The primary reviewer (author MAC) conducted the searches and downloaded the results of each database into a separate file on a records management software (EndNote X9). A combined file was created at the end of the search and duplicate entries were removed.

The primary reviewer screened the studies' titles and abstracts and selected the studies that matched the inclusion criteria, followed by the screening of full-text records. The second reviewer screened the titles and abstracts of a random selection of 250 records and 25 full text records (which amounted to over 10% of full text studies identified). Consensus regarding inclusion was reached for all records after discussion between the reviewers and the senior authors. The number of studies excluded and the reasons for exclusion of full text studies is documented and reported in Figure 1.

Data extraction process:

A data extraction form was developed by the primary reviewer (MAC) and was piloted by the two reviewers with 4 studies. The pilot was refined until a final data extraction form was agreed by all co-authors. The secondary reviewer performed an independent check of the data extraction in all studies included in the review.

Data were extracted on study characteristics including sample size and characteristics; type of healthcare setting; and country. Using meta-ethnography methods, data were collected on first- and second-order constructs.

Data synthesis

Data were synthesised using meta-ethnography. This method was chosen as meta-ethnography aims to arrive at a higher order of interpretation, with the potential of going

For peer review only - http://bmjopen.bmj.com/site/about/guidelines.xhtml

beyond a synthesis of the original studies to generate new knowledge on a topic, while maintaining the integrity of interpretations from the primary studies.

Meta-ethnography treats the views of study participants as 'first-order constructs' and the explanations of each study's authors as 'second-order constructs'. The reviewers sought to identify and synthesise the first- and second- order constructs, which were similar across studies in a process called 'reciprocal translation'[20]. The relationships that reviewers identified between findings from the different primary studies were treated as 'third-order constructs'[20]. The reviewers looked for contradictory findings within and between studies (intra- and inter-study contradictions) via the process of refutational translation and examined whether there were plausible explanations for these apparent contradictions, such as different contextual factors between studies[20 21].

Meta bias(es)

Risk of selection bias:

As described in the selection process, the reviewing team included a secondary reviewer, who conducted screening of a proportion of the search results separately. This method aimed to minimise the risk of bias arising from misclassification of studies as eligible/ineligible for inclusion in the review[22].

Risk of reporting bias:

The meta-ethnography methodology addresses risk of bias in the reporting of studies by separately considering the expressed views of participants and those of the study authors, as described in the Data Synthesis section.

Confidence in cumulative evidence

We used the GRADE-CERQual (Confidence in the Evidence from Reviews of Qualitative Research) approach[23-29] to assess the confidence in the review findings.

Risk of bias in individual studies

The studies included had their quality appraised independently by both reviewers. There was full agreement between the reviewers about the quality appraisal scores for all studies.

Quality assessment was based on Harden et al. (2009)[30], who suggest 12 criteria based on the quality of reporting, use of strategies to increase reliability and validity, and the extent to which the findings reflected participants' views and experiences. Studies were classified

For peer review only - http://bmjopen.bmj.com/site/about/guidelines.xhtml

as high quality (those meeting 10 or more criteria out of 12), medium quality (those meeting between seven and nine criteria) and low quality (those meeting fewer than seven criteria). Due to the current lack of consensus in the literature regarding quality assessment of qualitative studies[31 32], the quality assessment was not used to exclude studies from the review. Please see Appendix 2 for the details on the quality assessment of each study.

Reflexivity

All authors are female and based in the UK. The authors' professional backgrounds are varied and include clinical and academic psychiatry (MAC and LMH), undergraduate medical school (SB), academic medical sociology (HL) and applied health research (SO). Authors MAC and LMH also have direct experience of assessing and treating mental health service users who have been subject to, or perpetrated, DVA; MAC as a liaison psychiatrist based at a general acute hospital and LMH as a perinatal psychiatrist.

All authors believe that the evidence for the need for healthcare services to address gender-based violence is compelling, and that evidence on identifying and addressing DVA perpetration in healthcare is crucial. The lead author (MAC) explicitly considered how her professional background and previous experiences of assessing and treating DVA perpetrators may have influenced the research process. She reflected on previous clinical experiences and discussed those with her co-authors during the data collection and analysis, so as to limit the influence of professional or personal biases in the conduct of the research.

RESULTS

A total of 229 abstracts were identified as potentially meeting the inclusion criteria during title and abstract screening. Following full-text screening (see Fig. 1), six studies were included in the review; four of these studies were published in peer-reviewed journals, one in a published report and one is a doctoral thesis.

Studies reported data from 125 participants who met the review criteria, all but one of whom were adult males (see Table 2). All study participants were adults; other socio-demographic information, e.g., ethnicity, was not included in all papers. Five of the included studies focused on intimate partner violence (IPV); only Van Rooij et al. (2013)[33] included perpetrators of family violence (FV) as well as IPV perpetrators. One study[34] focused on men in same-sex relationships while the other 5 studies either focused on heterosexual relationships or did not mention the type of intimate partner relationships.

For peer review only - http://bmjopen.bmj.com/site/about/guidelines.xhtml

Types of healthcare setting included general practice[35]; community addictions service[36]; sexual health clinic[34] and community forensic psychiatry[33]; two studies[37 38] explored participants' experiences of healthcare without specifying the setting. Half of the included studies (n=3) recruited participants from 'community', non-forensic settings[34-36], and the remaining three studies recruited perpetrators of DVA from forensic settings, including perpetrator programmes[37], the criminal justice system[33] or prison[38]. For the latter group of studies, the constructs included in the analysis of this review related to experiences of use of healthcare services (including forensic psychiatry) but not of perpetrator programmes (see exclusion criteria). Four studies were conducted in the UK, one in the Netherlands[33] and one in North America[38].

All studies included individual qualitative interviews as the method of data collection; some studies also collected quantitative data, which were not analysed for this review. The three studies that reported the qualitative analysis methods[33 35 38] applied grounded theory[39]; the remaining three studies did not specify the data analysis methods used. The quality of reporting was assessed as medium in five of the six studies and as low in one.

Data for analysis were extracted from the full primary studies by the first author (MAC) following repeated reading of each individual study and independently checked by the second reviewer (SB) before being added to NVivo for coding[19]. Coding was independently checked by co-authors SO and HL; codes and themes were discussed by the authors and refinements were made where necessary.

Data were coded according to whether they originated from direct participant quotes (first order constructs) or from researchers' interpretations (second order constructs); and according to the themes or sub-themes they represented at the initial stage of analysis. After the initial coding stage, further rounds of coding and re-arranging of concepts and themes were conducted following discussions between the authors, until the final themes for first- and second-order constructs were identified. Third-order constructs were identified via an iterative process of re-reading the first- and second-order constructs and the full papers, translating the papers into each other by comparing the meaning of themes and their relationships across studies, and developing an over-arching model or 'line of argument synthesis' for the phenomenon studied. This was achieved by successive discussions between the authors. The context of each study, including characteristics of population and setting, and their strengths and limitations were taken into account in the interpretations.

Table 2. Study characteristics

For peer review only - http://bmjopen.bmj.com/site/about/guidelines.xhtml

Study	Setting	Country	Sample size and characteristics	Community or forensic sample	Quality appraisal score (please see Appendix 2 for details)
Hester et al. 2006[37]	Voluntary perpetrator programmes	UK	62 male IPV perpetrators	Mixed	Low (5/12)
Van Rooij et al 2013[33]*	Community forensic psychiatry	Netherlands	9 male and 1 female perpetrators of IPV and FV	Forensic	Medium (8/12)
Morgan et al 2014[35]*	General practice	UK	8 male IPV perpetrators	Community	Medium (9/12)
Swogger 2016[38]	Prison	US	15 male IPV perpetrators	Forensic	Medium (9/12)
Bacchus et al 2018[34]*	Sexual health clinic	UK	10 male IPV perpetrators in same sex relationships**	Community	Medium (7/12)
Hashimoto et al 2018[36]	Addiction services	UK	20 male IPV perpetrators	Community	Medium (7/12)

*these studies also included other participants who were not perpetrators of DVA. Data from those participants was not included in the analysis.

** these participants disclosed perpetration of at least one abusive behaviour towards a partner in a survey and/or in the qualitative interviews, but not all described themselves as 'IPV perpetrators'

First order constructs

Analysis of the 6 qualitative papers identified 30 first order constructs, which were grouped into 6 key themes (see Table 3; participants' emotions towards DVA, attitudes towards DVA; experiences of healthcare use; triggers to wanting to instigate behavioural

For peer review only - http://bmjopen.bmj.com/site/about/guidelines.xhtml

changes with regards to DVA perpetration; attitudes towards enquiry about DVA by healthcare services' staff; and concerns about confidentiality.

Emotions about DVA perpetration or relationship difficulties

Some participants described experiencing shame, embarrassment and regret over their abusive behaviours towards intimate partners and reported to researchers that those emotions meant they were unwilling to admit to those behaviours to clinicians[36]. Other participants reported feeling blamed by healthcare services when disclosing DVA perpetration, particularly when they had also experienced DVA in their relationships and when the abuse led to the involvement of child protection services[38].

Attitudes towards DVA perpetration or relationship difficulties

Some participants minimized the importance of DVA, placing more emphasis on the positive aspects of their intimate relationship. Others normalised DVA, reporting views that abusive behaviours were an expected part of intimate relationships, rather than something that required specific help or intervention[36], indicating lack of awareness of what constitutes abuse in a relationship. Participants who reported that other issues in their lives (such as housing or financial problems) took priority over their relationship problems voiced that they were less likely to disclose relationship difficulties to healthcare professionals or to seek any type of support for those difficulties[33,36], reflecting a low prioritisation of DVA among other stressors.

Experiences of healthcare use

Some participants described how it took them many years to decide to seek help for DVA perpetration. Some experienced a long and convoluted route to access the support they wanted via healthcare services[36 37], while others did not seek help from healthcare professionals as they believed healthcare staff lacked the knowledge or expertise to address the perpetration of abusive behaviours[36]. When participants decided to seek help from healthcare professionals for relationship problems, some specifically wanted a referral for an 'anger management' programme[36 37]. A range of different services were accessed, e.g., addictions services, general practice and mental health services.

Experiences of psychological support were mixed, with some participants describing how individual or couples' therapy had been ineffective in reducing their use of abusive behaviours, or how they had felt blamed for their relationship problems within therapy[38]. Couples' counselling was perceived as unhelpful in changing perpetrators' behaviour when the intervention was focused on the victim's experiences rather than on the perpetrator's

For peer review only - http://bmjopen.bmj.com/site/about/guidelines.xhtml

experiences and behaviour [38]. A negative experience mentioned by participants in the study focusing on men in same-sex relationships included when they thought that their general practitioners did not know enough about their personal life, for example about their sexuality, to allow disclosure of relationship problems[34].

Participants in the studies set in prisons and in community forensic psychiatry, however, described how they were able to establish a close rapport with an individual healthcare worker who listened to them, or that they were pleased to gain access to psychotherapeutic interventions to focus on addressing their abusive behaviours[33 38]. Practical support, such as help with public transport fares to attend appointments, was appreciated. In one case, healthcare use led to access to professional reports that assisted in the participant's court case related to DVA[33]. Another attributed positive change to a combination of psychiatric treatment and a perpetrator programme undertaken while serving a prison sentence for DVA[38]; this is described in more detail in the following theme below.

Triggers that instigate behavioural changes with regards to DVA perpetration

Participants articulated how escalation of abuse, relationship breakdown, fear of losing contact with their children, being arrested or suffering other consequences of their behavior (such as being banned from social spaces) acted as important triggers to disclosing DVA perpetration and to seeking help via healthcare services[35 37]. Some participants viewed those events as 'wake-up calls' to come to terms with their behaviour and seek help. Engagement with mental health care and programmes for reduction of violent behaviour offered by prison healthcare services appeared to be a catalyst for change for one participant, who reported positive changes such as increased self-awareness and feeling less overwhelmed by his emotions through treatment[38].

Attitudes towards enquiry about DVA by healthcare services' staff

Participants' views about the acceptability of enquiry about DVA varied. In the study set in general practice, some thought that targeting enquiry only to individuals that show signs associated with DVA (e.g., physical marks or emotional distress) was more acceptable than universal enquiry in that setting[35]; nevertheless, one participant (who had disclosed perpetration of physical, psychological and verbal abuse against a partner in the research questionnaire) was completely opposed to any questions about DVA, as he perceived DVA or relationship problems as an entirely private matter[35].

In the sexual health study, selective enquiry was also mentioned as preferable to universal enquiry by one participant; another participant was in favour of enquiry, but suggested that the initial assessment was the ideal time for it. Both participants felt that that an

For peer review only - http://bmjopen.bmj.com/site/about/guidelines.xhtml

explanation about the link between DVA and the health problems addressed by that service would help individuals understand the rationale for, and feel more comfortable with, the question. In the same study, the provision of emotional support from a counsellor or non-medical health advisors was also mentioned as a condition for the acceptability of enquiry about DVA[34].

Concerns about confidentiality

Concerns about confidentiality acted as a key barrier to disclosure of relationship problems to clinicians[34 36]. Participants in the study set in an addictions clinic feared the potential consequences of disclosure, such as involvement from children's social services. One participant described that, for him, it was preferable to endure the consequences of being in a mutually abusive relationship than to seek help, due to his fear of losing the custody of his children if his relationship problems became known[36].

Having information about DVA documented in their general practice medical records was also a concern for some; other services, such as sexual health clinics, were perceived to offer more privacy and anonymity than primary care[34].

Table 3. First-order constructs

**Themes**	**First order constructs**
Emotions towards DVA perpetration	Shame, embarrassment, regret[36]<br>Feeling blamed[38]
Attitudes towards DVA perpetration	Normalisation, minimization, low prioritization of relationship problems[33 36]
Experiences of healthcare use	Positive experiences:<br>good rapport with healthcare workers;<br>feeling listened to;<br>accessing interventions that address abusive behaviours[33 38];<br>practical support[33]<br><br>Negative experiences:<br>feeling blamed by healthcare services[38];<br>couples' therapy focusing on the victim;

For peer review only - http://bmjopen.bmj.com/site/about/guidelines.xhtml

	delays and difficulties in accessing support[36 37],<br>believing that healthcare workers lack expertise on DVA[36] or do not know enough about service users' personal lives[34]<br><br>Referring to 'anger' as a way of disclosing DVA[36 37]
Triggers to change	Escalation of abuse; relationship breakdown[35 37]<br><br>Fear of loss of contact with children[37]<br><br>Social consequences, including being arrested or being banned from social spaces[35 37]<br><br>Positive engagement with healthcare services[38]
Attitudes towards enquiry about relationship problems	Enquiry is more acceptable at the initial assessment of an individual by a healthcare service[34], and if it is linked with the healthcare problems for which that individual is seeking help[34 35]<br><br>Availability of emotional support is a necessary element to allow enquiry about relationship problems[34]
Concerns about confidentiality	Fear of children's social services' involvement[34 36]<br><br>Perception that certain services (e.g., sexual health clinics) are more private than general practice[34]

Second order constructs:

For peer review only - http://bmjopen.bmj.com/site/about/guidelines.xhtml

Thirty second order constructs were identified; these were grouped in nine themes. Of those, six themes were duplications of the themes that emerged from the first order constructs (emotions towards DVA; attitudes around DVA; experiences of healthcare use; triggers to wanting to instigate behavioural changes with regards to DVA perpetration; attitudes towards enquiry about DVA by healthcare services' staff; and concerns about confidentiality); those will not be reported in this section. Three new themes emerged that had not appeared in first order constructs: the help-seeking journey; healthcare services' ability to address DVA; and under-reporting of relationship difficulties (see Table 4).

Help-seeking journey

Researchers in the study set in perpetrator programmes described how participants accessed healthcare services through different routes: a reactive route, following contact with other services (often the police or social services) due to their abusive behaviour, or a proactive route, when individuals sought help directly through healthcare for their relationship problems or a health problem related to their abusive behaviours. Researchers interpreted that individuals who sought help proactively and were explicit about their use of abusive behaviours found the help they desired more easily. Usually this was in the form of accessing a treatment programme for DVA perpetrators or another intervention directly addressing abusive behaviour[37].

Some individuals sought help for their relationship problems in indirect ways, by reporting anger[36 37] or common mental health problems such as depressive symptoms[37]. These participants reported finding it unhelpful when doctors responded to this type of help-seeking by prescribing antidepressant medication or referring service users to general counselling services without exploring the reasons behind their symptoms.

Some participants hoped to access specific support to reduce abusive behaviours, often framed as 'anger management services', and were frustrated when those services proved difficult to find or to access. Researchers proposed that the ability to access services promptly was crucial, as individuals were often ambivalent towards behavioural change and might have changed their minds about help-seeking if there was a long wait to access a service[37].

Healthcare services' ability to address DVA

Researchers suggested that participants' willingness to discuss relationship difficulties with healthcare staff in the addictions and sexual health settings were affected by what they perceived to be limitations of healthcare services[34 36].

For peer review only - http://bmjopen.bmj.com/site/about/guidelines.xhtml

In the addictions setting, researchers identified that participants were aware of time constraints within services and decided to use their consultations to discuss problems other than their relationship difficulties. Professionals' lack of expertise to address relationship problems was also mentioned by addiction service users as a barrier to disclosure, though it was not clear to the researchers how that perception had developed, and whether it had originated in participants' beliefs about the appropriateness of discussing relationship problems in addictions services. It was noted by the researchers that many participants reported that they had never been asked about their relationships by clinicians in that setting[[36]].

Concerns about clinicians' lack of time or skills to address relationship difficulties were also identified by researchers in the study located in a sexual health clinic: participants suggested that the sexual health clinic was a more appropriate venue for inquiry and disclosure of relationship problems than primary care, which was perceived as a setting where staff did not have the skill or time to explore issues about domestic abuse. Sexual health clinics were also seen by participants in that study as a more 'private' setting where sensitive and intimate issues could be discussed. Researchers recommended training for staff in healthcare services to increase awareness of DVA and improve their communication skills to help build trusting relationships with service users[[34]].

Under-reporting of relationship difficulties

Some participants under-reported DVA perpetration; researchers' interpretations included lack of recognition of what constitutes DVA[[34]], minimization[[36 38]], shame and fear of the consequences of disclosure[[34]], including loss of contact with one's children through social services intervention[[36]].. In two studies, participants denied having ever perpetrated abuse, despite having been convicted of DVA-related offences or having answered affirmatively to questions about DVA perpetration in the quantitative survey in the same study[[36 38]]. Researchers concluded that the denial or lack of awareness indicated a mismatch between actions (even those that participants had admitted to) and perception of what constitutes DVA, which may also contribute to the tendency towards under-reporting of DVA perpetration to healthcare professionals[[34 36]] in addition to other factors, such as shame and fear of the adverse consequences of disclosure.

Table 4. Second-order constructs

Themes	Second order constructs
Help-seeking journey	Reactive vs. proactive help-seeking[[37]]

For peer review only - http://bmjopen.bmj.com/site/about/guidelines.xhtml

	Implicit vs. explicit help-seeking[36 37]
Healthcare services' ability to address DVA	Healthcare services' time constraints and competing priorities[34 36]<br><br>IPV 'not a legitimate issue' to discuss with healthcare staff[36]<br><br>Lack of relevant expertise by staff[34 36]
Under-reporting of relationship difficulties	Minimisation of DVA[36 38]<br><br>Fear of consequences[34 36]<br><br>Shame regarding DVA[36]<br><br>Lack of recognition of what constitutes DVA[34 36]

Third-order constructs:

The constructs identified in these studies were suited for the creation of a *line or argument synthesis*, in which studies can be synthesized when they identify different aspects of a phenomenon which can be integrated to provide a new interpretation. Other possible approaches under the meta-ethnography method were reciprocal synthesis or refutational synthesis. Although certain themes were present in a number of studies, during analysis it became evident that the studies approached different aspects of DVA perpetrators' healthcare experiences, which complemented one another, making a line of argument synthesis more appropriate than reciprocal synthesis, There were no major or unexplained contradictions within studies or between studies, making the data not suitable for a refutational synthesis.

The third order constructs originated from this meta ethnography can be divided in factors that facilitate DVA perpetrators' disclosure of relationship difficulties to healthcare staff and their engagement with healthcare services; and factors that act as barriers to disclosure or DVA or healthcare service engagement (see Table 5).

For peer review only - http://bmjopen.bmj.com/site/about/guidelines.xhtml

Factors that facilitate DVA perpetrators' disclosure of DVA to healthcare staff and help-seeking

Some individuals reported having explicitly requested help to reduce abusive behaviours, in some cases triggered by a "crisis" (such as separation from a partner, escalation of abuse, or arrest), and sought or expected a specific intervention to address their problems. Others hinted about abuse when speaking to clinicians, referring to 'relationship problems', 'anger' or reporting low mood. Active listening by professionals, further exploration of relationship history, skills in enquiring about relationship difficulties sensitively and linking these to service users' health concerns tended to facilitate engagement of DVA perpetrators with healthcare services. The availability of on-site support for relationship problems may also act as a facilitator and may be a necessary condition for some to disclose their relationship problems. It was unclear what form this immediate support needs to take beyond non-judgmental listening by professionals or practical support.

Factors that act as barriers to disclosure of DVA and engagement with healthcare services

The studies demonstrated that perpetrators' perceptions that healthcare professionals lack expertise to address DVA and their own low prioritization of relationship problems negatively affect willingness to disclose relationship difficulties to healthcare staff.

Some individuals feel shame, guilt or regret when discussing abusive behaviours with healthcare staff, whereas others see abusive behaviours as a normal part of an intimate relationship, or did not recognize their own behaviours as abusive. These factors affect whether (and how) individuals seek help. Concerns about being blamed for relationship problems and about confidentiality, particularly due to fears of involvement from other services (e.g., children's social services), also act as important barriers to disclosure of DVA to healthcare professionals. These concerns, combined with negative emotions and attitudes towards DVA, are some of the factors underlying the under-reporting of DVA by perpetrators.

Table 5. Third order constructs

Third order construct	Description
Facilitators of disclosure of DVA to healthcare staff and engagement with healthcare	Reaching a crisis point or experiencing negative social consequences following abusive behaviour<br><br>Active listening by healthcare professionals

For peer review only - http://bmjopen.bmj.com/site/about/guidelines.xhtml

	Availability of emotional and practical support (ideally on-site)
Barriers to disclosure of DVA to healthcare staff and engagement with healthcare	Negative emotions and attitudes towards DVA by perpetrators<br><br>Lack of recognition of what constitutes DVA<br><br>Fear of consequences of disclosure<br><br>Lack of trust in healthcare services' knowledge or expertise in addressing DVA

Line of argument synthesis

The third-order constructs identified in this review have been integrated to propose a model for how perpetrators of DVA experience and engage with healthcare services. DVA perpetration is a complex phenomenon that can be experienced differently by individuals, and many perpetrators do not identify as such. An intra-study contradiction was identified involving the constructs of attitudes and emotions related to DVA. Some participants expressed shame and regret over their behaviours, while others minimised or normalised DVA[[36]]. The minimization and normalisation are consistent with the findings of a recent qualitative study of male IPV perpetrators in treatment for addiction and their female partners, which examined the perspective of perpetrators and their partners on the role of substance use in IPV[[40]]. In that study, perpetrators tended to attribute IPV to isolated events triggered by specific disputes, while their partners described a pattern characterized by enduring abuse, including severe violence and coercive control in some cases. The intra-study contradiction identified in our review highlights how individual emotional reactions to DVA by perpetrators can vary, with emotions ranging from denial and normalisation to self-reflection, shame and regret over their actions.

The journey to disclosure of perpetration of abusive behaviours to healthcare services is heterogenous and can start with an individual moving from a position of not identifying themselves as a perpetrator of DVA, to accepting that label due to reaching a point of crisis with wider social consequences such as being banned from certain spaces or being sentenced by the criminal justice system. This, however, is not the case for all individuals who perpetrate DVA; some will remain unwilling to disclose abusive behaviours to healthcare staff due to strong negative emotions and attitudes towards DVA perpetration,

For peer review only - http://bmjopen.bmj.com/site/about/guidelines.xhtml

including denial, minimization and possibly a lack of understanding of what constitutes abuse.

When individuals had already experienced the adverse consequences of perpetrating abusive behaviours, such as relationship separation, escalation of abuse, or exclusion from social spaces, those events acted as triggers to disclosure and help-seeking. In these situations, perpetrators had decided to disclose their difficulties to clinicians to seek help and avoid further negative consequences. Those who were able to be more explicit about their problems to healthcare staff had a more positive experience of the support received, indicating that openness about the use of abusive behaviours and ability to discuss them with a healthcare professional in an environment perceived as non-judgmental can be the start of a positive engagement with healthcare services.

From the perspective of healthcare services, there are challenges in engaging with DVA perpetrators due to the barriers outlined above, and due to the perception by some individuals that healthcare services are not the right place to discuss DVA, or lack the expertise to understand the problem and provide the necessary support. Healthcare services have to tread a fine line between engaging individuals using a non-judgmental approach while also maintaining the safety of others, including children, which will often require breaking patient confidentiality. Encouragingly, some DVA perpetrators think that interventions by healthcare staff, such as providing active listening and emotional or practical support, can help, though it was not clear what specific support was desired beyond referrals to interventions such as anger management programmes.

Confidence in review findings: GRADE-CERQual assessment

The GRADE-CERQual assessment was applied to all included studies, as shown in Table 6. The assessment identified that all studies included were relevant to the review questions and populations. Four out of 6 studies included only male perpetrators of IPV in heterosexual (or not otherwise specified) relationships, while one study included perpetrators of IPV and FV (including one female) and another study included only male perpetrators of IPV in same-sex relationships. All of these are relevant to the review questions, though the implications of their specific findings will be discussed later in this paper. Minor to moderate concerns with regards to methodological limitations were identified, due to studies not reporting the methods used to establish the validity of data collection tools, data analysis methods, and/or quantity or richness of data in most study findings; no findings presented a high level of concern. Due to the small number of studies and participants, we were unable to assign a high level of confidence to any of the findings, although there was a good level of coherence between study findings (considering the

For peer review only - http://bmjopen.bmj.com/site/about/guidelines.xhtml

small study numbers) with each third-order construct being anchored by at least one third of the included studies.

Table 6: GRADE-CERQual assessment

**Review findings**	**Studies contributing**	**Methodological limitations**	**Relevance**	**Coherence**	**Adequacy of data**	**CERQual assessment**
Reaching a crisis point or experiencing negative social consequences following abusive behaviour	Morgan et al., 2014 [35]; Hester et al., 2006[37]	Minor concerns regarding data collection and analysis (Morgan et al., 2014[35]) Moderate concerns regarding reporting of recruitment/sampling, data collection and analysis (Hester et al., 2006[37])	Relevant (both studies)	2/6 studies There was one inter-study contradiction explained by the context of the perpetrators' experiences and the timing of help-seeking.	Minor concerns over quantity (Morgan et al 2014[35]) and richness of data (Morgan et al 2014[35]; Hester et al 20016 [37])	Moderate confidence (minor concerns over adequacy of data and methodological limitations)
Active listening by healthcare professionals	Van Rooij et al., 2013[33]; Swogger 2016[38]	Minor concerns regarding data collection and analysis (both studies)	Relevant (both studies)	2/6 studies	Moderate concerns over richness (van Rooij et al 2013[33]) and quantity of data (Swogger et al	Moderate confidence (moderate concerns over adequacy of data and minor concerns over methodological limitations)

For peer review only - http://bmjopen.bmj.com/site/about/guidelines.xhtml

					2016[38]; van Rooij et al 2013[33])	
Availability of emotional and practical support (ideally on-site)	Van Rooij et al., 2013[33]; Swogger 2016[38]; Bacchus et al., 2018[34]	Minor concerns regarding data collection and analysis (all 3 studies)	Relevant (all 3 studies)	3/6 studies	Moderate concerns regarding quantity of data (Swogger et al 2016[38]; van Rooij et al 2013[33]) Minor concerns over richness of data (Bacchus et al, 2018[34])	Moderate confidence (moderate concerns over adequacy of data and minor concerns over methodological limitations)
Negative emotions and attitudes towards DVA by perpetrators	Van Rooij et al., 2013[33]; Hashimoto et al., 2018[36]; Swogger 2016[38]	Minor concerns regarding data collection and analysis (all 3 studies)	Relevant (all 3 studies)	3/6 studies There was one intra-study contradiction explained by variation in individual emotional reactions to DVA by perpetrators	Moderate concerns regarding quantity (Swogger 2016[38]; van Rooij et al. 2013[33]) and richness of data (van Rooij et al, 2013[33])	Moderate confidence (moderate concerns over adequacy of data in 2 out of 3 studies, and minor concerns over methodological limitations in all 3 studies)

For peer review only - http://bmjopen.bmj.com/site/about/guidelines.xhtml

					No concerns (Hashimoto et al., 2018[36])	
Fear of consequences of disclosure	Hashimoto et al., 2018[36]; Bacchus et al., 2018[34]	Minor concerns regarding data collection and analysis (both studies)	Relevant (both studies)	2/6 studies	No concerns (Hashimoto et al, 2018[36]) Moderate concerns regarding quantity and richness of data (Bacchus et al, 2018[34])	Moderate confidence (moderate concerns over adequacy of data in one study, and minor concerns over methodological limitations in both studies)
Lack of trust in healthcare services' knowledge or expertise in addressing DVA	Hashimoto al., 2018[36]; Bacchus et al., 2018[34]; Swogger 2016[38]	Minor concerns regarding data collection and analysis (all 3 studies)	Relevant (all 3 studies)	3/6 studies	Minor concerns over quantity of data (all 3 studies)	Moderate confidence Moderate confidence (minor concerns over adequacy of data and methodological limitations)

DISCUSSION

Main findings

Our findings indicate that the experiences of healthcare use by DVA perpetrators are complex, and may depend on whether individuals self-identify as perpetrators of abusive behaviours and what stage they are in their help-seeking journey. When healthcare

For peer review only - http://bmjopen.bmj.com/site/about/guidelines.xhtml

For peer review only

services provide a listening space and offer emotional and practical support to those who are ready to indicate (even if tentatively) that there are problems in their relationships, individuals feel more inclined to disclose abusive behaviours to clinicians.

The barriers to the disclosure of abusive behaviours by perpetrators to healthcare workers include feelings of shame, beliefs that abuse is a normal part of intimate relationships (or a lack of recognition of what constitutes abuse), concerns about confidentiality and a lack of trust in healthcare professionals' ability or readiness to address DVA. The latter finding, in particular, is consistent with the experiences of healthcare professionals regarding their readiness to address DVA in their practice, as identified by a recent systematic review[41],

Overall, it is important to recognize that healthcare services need to navigate the experiences and preferences of DVA perpetrators while maintaining safe professional practice and safeguarding survivors of abuse. One key barrier to disclosure of perpetration of abusive behaviours was the concern about confidentiality and the desire to avoid negative social consequences, such as the perceived threat of losing contact with one's children via involvement of children's social services[36]. Given the risk posed by DVA to children[8-10] and criticism made of services regarding poor inter-agency working and information-sharing in cases of domestic homicides[42], it would be inappropriate and unsafe for healthcare staff not to share information pertaining to the physical or emotional safety of children with other agencies such as social services or (when appropriate) police. It is, therefore, a challenge for healthcare services to establish ways in which to provide individuals with a listening space while being clear about the limits of confidentiality.

The study also found that some negative consequences of DVA perpetration, such as involvement of children's social services, being banned from social spaces, or relationship breakups may act as barriers or facilitators to disclosure to healthcare services. This apparent contradiction can be explained by the context of the perpetrators' experiences and the timing of help-seeking, as outlined in the previous section. Importantly, some of these factors, such as relationship breakdown, are known to be risk factors for domestic homicides[42 43], and are crucial for the assessment of risk by healthcare professionals.

In common with our findings, a recent systematic review on the healthcare experiences of DVA survivors[44] found that a positive relationship with a clinician and a safe and confidential environment acted as facilitators to disclosure of DVA, while fear of the consequences of disclosure and concerns that healthcare services may not be able to help acted as barriers to disclosure. Lack of recognition what constitutes abuse, or the normalisation of abuse have also been reported by studies conducted with survivors. However, a host of other themes identified in the survivors' studies, including concerns about personal safety, feelings of powerlessness, financial dependence on the perpetrator,

For peer review only - http://bmjopen.bmj.com/site/about/guidelines.xhtml

lack of social support, or avoidance of re-living trauma, were absent from studies involving perpetrators, demonstrating the vulnerability of survivors and the power imbalances characteristic of DVA[45].

All but one of the participants in the studies included were male, therefore some issues identified may be linked to patterns of help-seeking among men. It has been reported that men have lower rates of help-seeking for health problems[46], and a systematic review has described a number of barriers to help-seeking for health problems in men[47], some of these barriers were also present in our findings, such as embarrassment, fear and difficulty communicating with healthcare professionals. Traditional masculine ideologies may also be a factor in barriers to perpetrators of DVA, or more specifically IPV, to engaging with healthcare; a longitudinal study of male IPV perpetrators who used substance use services found that participants who espoused traditional masculine ideologies were less likely to engage with supporting services such as healthcare and employment or vocational support[48].

Strengths and limitations of this review

Our review provides the first systematic review and synthesis of qualitative research exploring the healthcare experiences of DVA perpetrators, addressing a significant gap in the current literature on DVA in healthcare services. We used a rigorous systematic review methodology, including comprehensive systematic search and the use of the GRADE-CERQual approach to assess the confidence in each review finding[49].

The *a priori* review questions were mostly answered: findings were able to address DVA perpetrators' experiences of discussing DVA with healthcare staff; their experiences of using healthcare services when their history of DVA perpetration is known; and their experiences of seeking support to address relationship difficulties were explored in the original studies and in the interpretive findings of this review. However, the review question regarding DVA perpetrators' views on the association between their health and relationship difficulties or abusive behaviours could not be addressed as it was not explored in any of the contributing studies.

One of the limitations of the review is the small number of studies found; twice as many studies were included in a meta-synthesis of the experiences of DVA survivors[50], indicating that DVA perpetration continues to be an under-researched topic relative to experiences of DVA. Most studies, with the exception of Hester et al., 2006[37] had a relatively small number of participants. It is important to recognize that, as well as being under-researched, the DVA perpetrator population may also be hard to reach due to its low identification in healthcare services, and there are challenges, including ethical ones, in

For peer review only - http://bmjopen.bmj.com/site/about/guidelines.xhtml

recruiting such populations. The healthcare experiences of DVA perpetrators were not the primary focus of most included studies and made up only a small proportion of the data presented. There was sufficient data to identify common themes between the studies and develop a 'line of argument' synthesis, but the availability of data limited the authors' ability to develop a new theory regarding the topic.

Studies included in the review were conducted in a variety of healthcare settings; this is a strength, in that the representation of different healthcare settings and clinical populations has helped address a variety of aspects of participants' healthcare experiences. However, this affects the generalizability of certain findings. One study, for example, included only individuals in same-sex relationships[34], whereas the other five studies either did not specify the type of relationship or included only individuals in heterosexual relationships[35-38] (or a mixture of intimate partner relationships and adult family relationships[33]). Certain findings, such as the importance of privacy in a healthcare encounter, were identified only in the study conducted with individuals in same-sex relationships, suggesting that stigma leading to identity concealment and other types of 'minority stress' associated with being in a same-sex relationship[51 52] may add an additional barrier to disclosure of DVA perpetration.

Another aspect of this review that is both a strength and a limitation is the fact that the majority of study participants were male perpetrators of IPV. This limits how the review findings can be generalized to the (significantly under-researched) population of perpetrators of FV, but it is a strength in that we can have higher confidence in the findings in relation to male IPV perpetrators.

This review did not identify any studies from non-Western, non-English speaking or low- and middle-income countries that met inclusion criteria, nor studies focusing on perpetrators of non-partner adult family violence or female perpetrators of DVA. This raises the question of whether the findings are generalizable to those populations.

Implications for clinical practice and policy

Our findings demonstrate some of the complexities associated with addressing DVA perpetration in healthcare services. Relationship difficulties and DVA can elicit a variety of reactions in perpetrators, some of which may be conflicting, and which affect the likelihood of disclosure of abusive behaviours to healthcare professionals. Additionally, individuals may present to healthcare services with the aim to seek help for the consequences of relationship difficulties (e.g., low mood) without disclosing DVA perpetration directly. This highlights the importance of a trusting rapport between healthcare professionals and

For peer review only - http://bmjopen.bmj.com/site/about/guidelines.xhtml

service users, as well as willingness on the part of clinicians to explore the intimate and family relationships of their service users in more detail.

Some of the participants of the studies included in this review articulated that DVA was not a legitimate issue to bring to consultations with healthcare professionals[36], while others stated that it seemed appropriate to discuss DVA with clinicians in services that were perceived as more 'private', such as sexual health services[34]. An assurance of privacy may be particularly important for individuals in same-sex relationships, as previously discussed. Moreover, some participants were not aware of what behaviours constitute abuse[34 36]. This suggests that, beyond raising awareness of DVA perpetration among healthcare professionals, it may be necessary to raise public awareness of what behaviours constitute perpetration of abuse. Public awareness campaigns on DVA, including the campaign by the UK Home Office during the COVID-19 pandemic[53], often address survivors or those at risk of experiencing DVA; campaigns directed at perpetrators will likely need a different language and will need to be circulated through different channels to address the populations at risk of perpetrating DVA. For those campaigns to be effective, the training of health professionals (and others who are likely to encounter DVA, such as the police and social care) on how to safely enquire about DVA perpetration and how to respond to disclosures will be essential.

A number of participants reported that they expected to receive support from healthcare services to access interventions to reduce their anger or violence ('anger management'), or a treatment programme for DVA perpetrators. Although a systematic review of qualitative studies has demonstrated that users of IPV perpetrator programmes find value in those interventions[54], the evidence for effectiveness of DVA perpetrator programmes in reducing risk to survivors is limited[55-57]; most intervention studies are restricted to specific populations or settings, making them less generalizable, and many lack a control group. However, psychological therapies such as behavioural couples' therapy (BCT) or cognitive behavioural therapy (CBT) combined with treatment for alcohol misuse can be effective in reducing abusive behaviours[57-60]. This suggests that the existing role of healthcare services in identifying and treating substance use may provide a route into addressing DVA perpetration when those issues co-exist.

CONCLUSION

Our results provide the first synthesis of evidence on the healthcare experiences of DVA perpetrators. Our meta-synthesis indicates that healthcare professionals need to be open to asking questions about service users' family and intimate relationships, and be attentive to requests for help that may not be explicit. These findings can inform the development of

For peer review only - http://bmjopen.bmj.com/site/about/guidelines.xhtml

training to improve the identification of DVA perpetration in healthcare settings. Our findings also suggest a possible role for public health campaigns directed at groups at risk of DVA perpetration to improve awareness of what constitutes abuse and encourage individuals to disclose those behaviours to healthcare professionals. DVA perpetration is a complex and multi-factorial that requires the involvement of a number of sectors, and further research is needed in how best to integrate the responses of healthcare with those of other agencies, such as social care, police and criminal justice system.

Figure legend:
Figure 1: PRISMA Diagram

Appendices:
Appendix 1: eMERGE checklist
Appendix 2: quality assessment

Contributhorship statement:

Dr Marilia A. Calcia: conceptualising the study; drafting and finalising review protocol; systematic literature search; primary reviewer for study selection and appraisal; meta-synthesis; wrote first draft of study for publication.

Simran Bedi: secondary reviewer for study selection and appraisal.

Professor Louise Howard, Dr Heidi Lempp, Dr Sian Oram: conceptualising the study; advising on methodology; supervision and editing of drafts.

Competing interests:

All authors have completed the ICMJE uniform disclosure form at www.icmje.org/coi_disclosure.pdf and declare: Professor Louise Howard and Dr Sian Oram receive salary support from the UKRI Cross-disciplinary Network on Violence, Abuse and Mental Health (www.vamhn.co.uk). Professor Louise M Howard also receives salary support from the NIHR SLAM/KCL Biomedical Research Centre and the NIHR South London Applied Research Collaboration. Dr Marilia A Calcia received salary support through a 3-month full time secondment to the Section of Women's Mental Health, Health Service and Population Research Department, IoPPN, KCL, from July-September 2018, from an NIHR Research Professorship to Louise Howard (NIHR-RP-R3-12-011). Dr Heidi Lempp, and Simran Bedi declare no financial relationships with any organisations that might have

For peer review only - http://bmjopen.bmj.com/site/about/guidelines.xhtml

an interest in the submitted work in the previous three years. The authors declare no other relationships or activities that could appear to have influenced the submitted work.

Ethical statement:

No ethical approval was sought as this study is a systematic review that analysed anonymised data from published studies, which have obtained informed consent/ethical approval.

Funding

Sponsors/funding: Dr Calcia is a self-funded MD(Res) student and received salary support through a 3-month full time secondment to the Section of Women's Mental Health, Health Service and Population Research Department, IoPPN, KCL, from July-September 2018, from an NIHR Research Professorship to Louise Howard (NIHR-RP-R3-12-011). Louise Howard is an NIHR Senior investigator. The funding organisations had no involvement in the study design; in the collection, analysis and interpretation of the data; in the writing of the report and in the decision to submit the study for publication.

Data availability statement:

The study involved analysis of anonymised participant data available in published research studies, a published report and an unpublished doctoral thesis. All studies included are referenced in the paper and are available online through research databases. DOIs or weblinks for the included studies are provided below.

van Rooij FB, Ten Haaf J, Verhoeff AP. Temporary restraining orders in the Netherlands: a qualitative examination of perpetrator and victim views. Journal of Family Violence. 2013 Jul 1;28(5):503-14. https://doi.org/10.1007/s10896-013-9520-2

Morgan K, Williamson E, Hester M, et al. Asking men about domestic violence and abuse in a family medicine context: Help seeking and views on the general practitioner role. Aggression and violent behavior. 2014 Nov 1;19(6):637-42. https://doi.org/10.1016/j.avb.2014.09.008

Hashimoto N, Radcliffe P, Gilchrist G. Help-seeking behaviors for intimate partner violence perpetration by men receiving substance use treatment: a mixed-methods secondary analysis. Journal of interpersonal violence. 2018 May 1:0886260518770645. https://doi.org/10.1177/0886260518770645

For peer review only - http://bmjopen.bmj.com/site/about/guidelines.xhtml

Bacchus LJ, Buller AM, Ferrari G, et al. “It’s Always Good to Ask”: a mixed methods study on the perceived role of sexual health practitioners asking gay and bisexual men about experiences of domestic violence and abuse. Journal of Mixed Methods Research. 2018 Apr;12(2):221-43. https://doi.org/10.1177/1558689816651808

Hester M, Westmarland N, Gangoli G, et al. Domestic violence perpetrators: identifying needs to inform early intervention. Newcastle: Northern Rock Foundation. 2006. https://aura.antioch.edu/etds/303/

Swogger R. Incarcerated Men and the Etiology of Intimate Partner Violence [PhD thesis]. Antioch University. 2016. http://aura.antioch.edu/cgi/viewcontent.cgi?article=1309&context=etds

For peer review only

References:

1. Organisation WH. Fact sheet: Violence against women. 2021
2. Office H. Domestic Abuse Bill 2020: overarching documents. Draft statutory guidance framework., 2020.
3. Elkin M. Domestic abuse in England and Wales: year ending March 2018. In: Statistics OfN, ed. https://www.ons.gov.uk/peoplepopulationandcommunity/crimeandjustice/bulletins/domesticabuseinenglandandwales/yearendingmarch2018, 2018.
4. Organization WH. Global and regional estimates of violence against women: prevalence and health effects of intimate partner violence and non-partner sexual violence: World Health Organization 2013.
5. Campbell JC. Health consequences of intimate partner violence. *The lancet* 2002;359(9314):1331-36.
6. Trevillion K, Oram S, Feder G, et al. Experiences of domestic violence and mental disorders: a systematic review and meta-analysis. *PloS one* 2012;7(12):e51740.
7. Howard LM, Oram S, Galley H, et al. Domestic violence and perinatal mental disorders: a systematic review and meta-analysis. *PLoS Med* 2013;10(5):e1001452.
8. Edleson JL. Children's witnessing of adult domestic violence. *Journal of interpersonal Violence* 1999;14(8):839-70.
9. Kitzmann KM, Gaylord NK, Holt AR, et al. Child witnesses to domestic violence: a meta-analytic review. *Journal of consulting and clinical psychology* 2003;71(2):339.
10. Flach C, Leese M, Heron J, et al. Antenatal domestic violence, maternal mental health and subsequent child behaviour: a cohort study. *BJOG: An International Journal of Obstetrics & Gynaecology* 2011;118(11):1383-91.
11. Oram S, Trevillion K, Feder G, et al. Prevalence of experiences of domestic violence among psychiatric patients: systematic review. *The British Journal of Psychiatry* 2013;202(2):94-99.
12. Yu R, Nevado-Holgado AJ, Molero Y, et al. Mental disorders and intimate partner violence perpetrated by men towards women: A Swedish population-based longitudinal study. *PLoS medicine* 2019;16(12):e1002995.
13. Rose D, Trevillion K, Woodall A, et al. Barriers and facilitators of disclosures of domestic violence by mental health service users: qualitative study. *The British Journal of Psychiatry* 2011;198(3):189-94.
14. Oram S, Capron L, Trevillion K. Promoting Recovery in Mental Health: Evaluation Report. *London: King's College London* 2016
15. Hester M, Ferrari G, Jones S, et al. Occurrence and impact of negative behaviour, including domestic violence and abuse, in men attending UK primary care health clinics: a cross-sectional survey. *BMJ open* 2015;5(5):e007141.
16. Moher D, Liberati A, Tetzlaff J, et al. Preferred reporting items for systematic reviews and meta-analyses: the PRISMA statement. *PLoS medicine* 2009;6(7):e1000097.
17. France EF, Cunningham M, Ring N, et al. Improving reporting of meta-ethnography: the eMERGe reporting guidance. *BMC medical research methodology* 2019;19(1):1-13.

For peer review only - http://bmjopen.bmj.com/site/about/guidelines.xhtml

18. Holdsworth E, Bowen E, Brown S, et al. Offender engagement in group programs and associations with offender characteristics and treatment factors: A review. *Aggression and Violent Behavior* 2014;19(2):102-21.
19. Seale C. Using computers to analyse qualitative data. *Doing qualitative research: A practical handbook* 2000:155-74.
20. Noblit GW, Hare RD. Meta-ethnography: Synthesizing qualitative studies: sage 1988.
21. Pilkington H. Employing meta-ethnography in the analysis of qualitative data sets on youth activism: a new tool for transnational research projects? *Qualitative Research* 2018;18(1):108-30.
22. Viswanathan M, Patnode CD, Berkman ND, et al. Assessing the risk of bias in systematic reviews of health care interventions. *Methods guide for effectiveness and comparative effectiveness reviews [Internet]* 2017
23. Booth A, Lewin S, Glenton C, et al. Applying GRADE-CERQual to qualitative evidence synthesis findings–paper 7: understanding the potential impacts of dissemination bias. *Implementation Science* 2018;13(1):63-70.
24. Colvin CJ, Garside R, Wainwright M, et al. Applying GRADE-CERQual to qualitative evidence synthesis findings—paper 4: how to assess coherence. *Implementation Science* 2018;13(1):33-41.
25. Glenton C, Carlsen B, Lewin S, et al. Applying GRADE-CERQual to qualitative evidence synthesis findings—paper 5: how to assess adequacy of data. *Implementation Science* 2018;13(1):43-50.
26. Lewin S, Bohren M, Rashidian A, et al. Applying GRADE-CERQual to qualitative evidence synthesis findings—paper 2: how to make an overall CERQual assessment of confidence and create a Summary of Qualitative Findings table. *Implementation Science* 2018;13(1):11-23.
27. Lewin S, Booth A, Glenton C, et al. Applying GRADE-CERQual to qualitative evidence synthesis findings: introduction to the series: BioMed Central, 2018.
28. Munthe-Kaas H, Bohren MA, Glenton C, et al. Applying GRADE-CERQual to qualitative evidence synthesis findings—paper 3: how to assess methodological limitations. *Implementation Science* 2018;13(1):25-32.
29. Noyes J, Booth A, Lewin S, et al. Applying GRADE-CERQual to qualitative evidence synthesis findings–paper 6: how to assess relevance of the data. *Implementation Science* 2018;13(1):51-61.
30. Harden A, Brunton G, Fletcher A, et al. Teenage pregnancy and social disadvantage: systematic review integrating controlled trials and qualitative studies. *Bmj* 2009;339
31. Daly J, Willis K, Small R, et al. A hierarchy of evidence for assessing qualitative health research. *Journal of clinical epidemiology* 2007;60(1):43-49.
32. Dixon-Woods M, Shaw RL, Agarwal S, et al. The problem of appraising qualitative research. *BMJ Quality & Safety* 2004;13(3):223-25.
33. van Rooij FB, Ten Haaf J, Verhoeff AP. Temporary restraining orders in the Netherlands: a qualitative examination of perpetrator and victim views. *Journal of Family Violence* 2013;28(5):503-14.
34. Bacchus LJ, Buller AM, Ferrari G, et al. "It's Always Good to Ask": a mixed methods study on the perceived role of sexual health practitioners asking gay and bisexual men about

For peer review only - http://bmjopen.bmj.com/site/about/guidelines.xhtml

experiences of domestic violence and abuse. *Journal of Mixed Methods Research* 2018;12(2):221-43.
35. Morgan K, Williamson E, Hester M, et al. Asking men about domestic violence and abuse in a family medicine context: Help seeking and views on the general practitioner role. *Aggression and violent behavior* 2014;19(6):637-42.
36. Hashimoto N, Radcliffe P, Gilchrist G. Help-seeking behaviors for intimate partner violence perpetration by men receiving substance use treatment: A mixed-methods secondary analysis. *Journal of interpersonal violence* 2018:0886260518770645.
37. Hester M, Westmarland N, Gangoli G, et al. Domestic violence perpetrators: identifying needs to inform early intervention. *Newcastle: Northern Rock Foundation* 2006
38. Swogger R. Incarcerated Men and the Etiology of Intimate Partner Violence. 2016
39. Corbin JM, Strauss A. Grounded theory research: Procedures, canons, and evaluative criteria. *Qualitative sociology* 1990;13(1):3-21.
40. Radcliffe P, Gadd D, Henderson J, et al. What role does substance use play in intimate partner violence? A narrative analysis of in-depth interviews with men in substance use treatment and their current or former female partner. *Journal of interpersonal violence* 2019:0886260519879259.
41. Hegarty K, McKibbin G, Hameed M, et al. Health practitioners' readiness to address domestic violence and abuse: A qualitative meta-synthesis. *PLoS one* 2020;15(6):e0234067.
42. Office H. Domestic homicide reviews: Key findings from analysis of domestic homicide reviews. 2016
43. Dawson M, Piscitelli A. Risk factors in domestic homicides: Identifying common clusters in the Canadian context. *Journal of interpersonal violence* 2017:0886260517729404.
44. Heron RL, Eisma MC. Barriers and facilitators of disclosing domestic violence to the healthcare service: A systematic review of qualitative research. *Health & Social Care in the Community*
45. Stark E. Coercive control. *Violence against women: Current theory and practice in domestic abuse, sexual violence and exploitation* 2013:17-33.
46. Wenger LM. Beyond ballistics: Expanding our conceptualization of men's health-related help seeking. *American journal of men's health* 2011;5(6):488-99.
47. Yousaf O, Grunfeld EA, Hunter MS. A systematic review of the factors associated with delays in medical and psychological help-seeking among men. *Health psychology review* 2015;9(2):264-76.
48. Wu E, El-Bassel N, Gilbert L, et al. Traditional male ideology and service system involvement among drug-involved men who perpetrate intimate partner violence: a longitudinal study. *Journal of interpersonal violence* 2011;26(8):1646-63.
49. Lewin S, Glenton C, Munthe-Kaas H, et al. Using qualitative evidence in decision making for health and social interventions: an approach to assess confidence in findings from qualitative evidence syntheses (GRADE-CERQual). *PLoS Med* 2015;12(10):e1001895.
50. Trevillion K, Hughes B, Feder G, et al. Disclosure of domestic violence in mental health settings: A qualitative meta-synthesis. *International Review of Psychiatry* 2014;26(4):430-44.

For peer review only - http://bmjopen.bmj.com/site/about/guidelines.xhtml

51. Longobardi C, Badenes-Ribera L. Intimate partner violence in same-sex relationships and the role of sexual minority stressors: A systematic review of the past 10 years. *Journal of Child and Family Studies* 2017;26(8):2039-49.
52. Rostosky SS, Riggle ED. Same-sex relationships and minority stress. *Current Opinion in Psychology* 2017;13:29-38.
53. Office H. Home Secretary's Statement on domestic abuse and coronavirus (COVID-19): 11 April 2020 In: Office H, ed., 2020.
54. McGinn T, McColgan M, Taylor B. Male IPV perpetrator's perspectives on intervention and change: A systematic synthesis of qualitative studies. *Trauma, Violence, & Abuse* 2020;21(1):97-112.
55. Eckhardt CI, Murphy CM, Whitaker DJ, et al. The effectiveness of intervention programs for perpetrators and victims of intimate partner violence. *Partner abuse* 2013;4(2):196-231.
56. NICE. Domestic violence and abuse: multi-agency working: National Institute for Health and Care Excellence (NICE) 2014.
57. Tarzia L, Forsdike K, Feder G, et al. Interventions in health settings for male perpetrators or victims of intimate partner violence. *Trauma, Violence, & Abuse* 2020;21(1):123-37.
58. Fals-Stewart W, Clinton-Sherrod M. Treating intimate partner violence among substance-abusing dyads: The effect of couples therapy. *Professional Psychology: Research and Practice* 2009;40(3):257.
59. O'Farrell TJ, Murphy CM, Stephan SH, et al. Partner violence before and after couples-based alcoholism treatment for male alcoholic patients: the role of treatment involvement and abstinence. *Journal of consulting and clinical psychology* 2004;72(2):202.
60. Satyanarayana VA, Nattala P, Selvam S, et al. Integrated cognitive behavioral intervention reduces intimate partner violence among alcohol dependent men, and improves mental health outcomes in their spouses: a clinic based randomized controlled trial from South India. *Journal of substance abuse treatment* 2016;64:29-34.

For peer review only

For peer review only - http://bmjopen.bmj.com/site/about/guidelines.xhtml

Figure 1: PRISMA Diagram

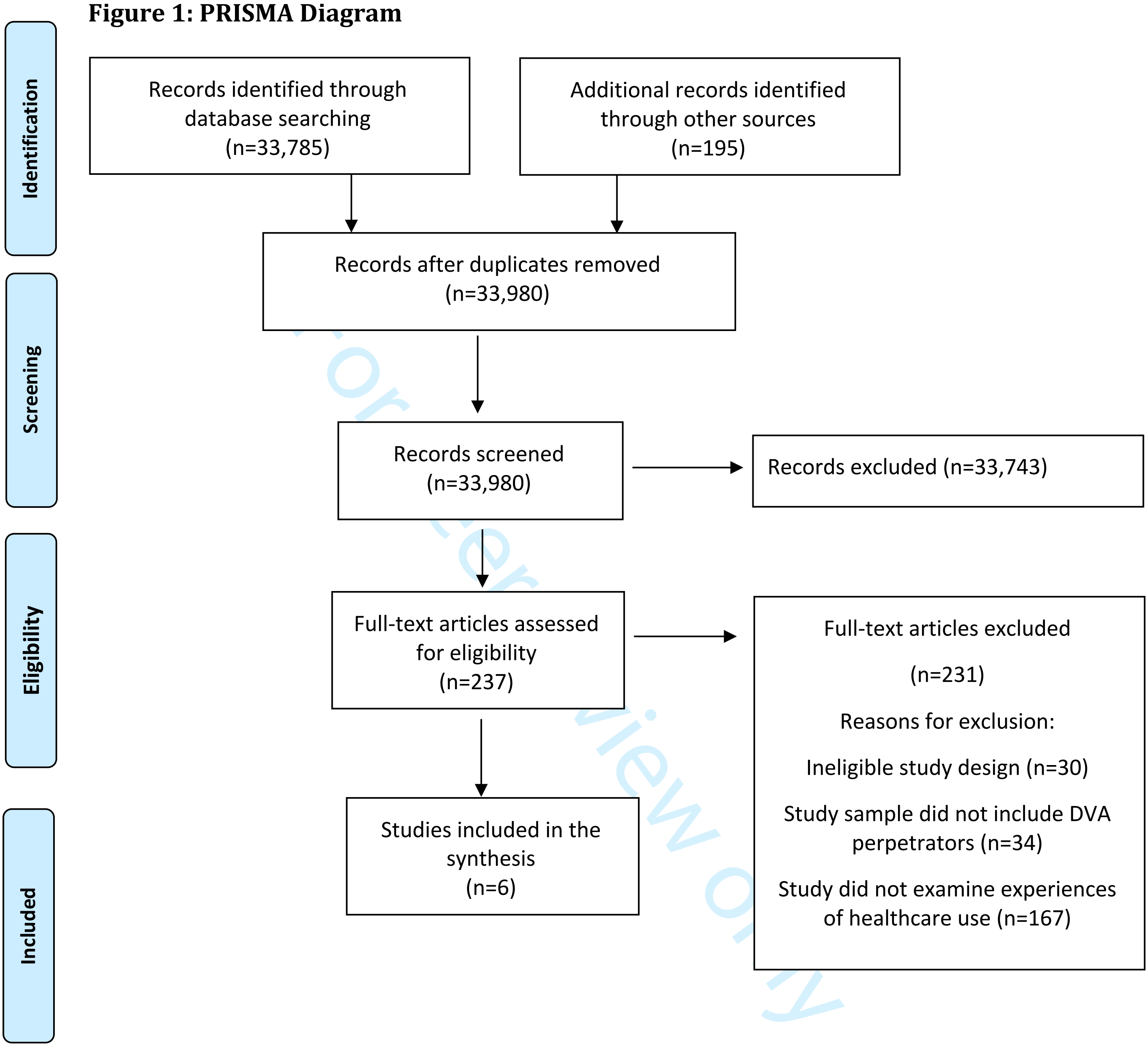

For peer review only - http://bmjopen.bmj.com/site/about/guidelines.xhtml

Table 1 The eMERGe meta-ethnography reporting guidance

From: Improving reporting of meta-ethnography: the eMERGe reporting guidance

Study: Calcia MA, Bedi S, Lempp H, Howard LM, Oram S. The healthcare experiences of perpetrators of domestic violence and abuse: a systematic review and meta-synthesis

For peer review only

No.	Criteria Headings	Reporting Criteria
Phase 1—Selecting meta-ethnography and getting started		
Introduction		
1	Rationale and context for the meta-ethnography	Describe the gap in research or knowledge to be filled by the meta-ethnography, and the wider context of the meta-ethnography<br><br>**Introduction (pages 3-5)**
2	Aim(s) of the meta-ethnography	Describe the meta-ethnography aim(s)<br><br>**Introduction (page 5)**
3	Focus of the meta-ethnography	Describe the meta-ethnography review question(s) (or objectives)<br><br>**Introduction (page 5)**
4	Rationale for using meta-ethnography	Explain why meta-ethnography was considered the most appropriate qualitative synthesis methodology<br><br>**Methods – Data synthesis (pages 8-9)**
Phase 2—Deciding what is relevant		
Methods		
5	Search strategy	Describe the rationale for the literature search strategy

No.	Criteria Headings	Reporting Criteria
		**Methods – Search strategy (page 6-7)**
6	Search processes	Describe how the literature searching was carried out and by whom<br><br>**Methods (pages 6-7)**
7	Selecting primary studies	Describe the process of study screening and selection, and who was involved<br><br>**Methods – Selection process (page 8)**
Findings		
8	Outcome of study selection	Describe the results of study searches and screening<br><br>**Results (pages 10-12)**<br>**PRISMA diagram (figure 1)**
Phase 3—Reading included studies		
Methods		
9	Reading and data extraction approach	Describe the reading and data extraction method and processes<br><br>**Methods (pages 8-9)**
Findings		
10	Presenting characteristics of included studies	Describe characteristics of the included studies<br><br>**Results (pages 10-12)**<br>**Table 2 (page 11)**
Phase 4—Determining how studies are related		
Methods		

For peer review only

No.	Criteria Headings	Reporting Criteria
11	Process for determining how studies are related	Describe the methods and processes for determining how the included studies are related:<br>- Which aspects of studies were compared<br>AND<br>- How the studies were compared<br><br>**Results (pages 11-21)**
Findings		
12	Outcome of relating studies	Describe how studies relate to each other<br><br>**Results (pages 19-24)**
Phase 5—Translating studies into one another		
Methods		
13	Process of translating studies	Describe the methods of translation:<br>- Describe steps taken to preserve the context and meaning of the relationships between concepts within and across studies- Describe how the reciprocal and refutational translations were conducted- Describe how potential alternative interpretations or explanations were considered in the translations<br><br>**Results and Discussion (pages 19-24)**
Findings		
14	Outcome of translation	Describe the interpretive findings of the translation.<br><br>**Results and Discussion (pages 19-26)**
Phase 6—Synthesizing translations		
Methods		
15	Synthesis process	Describe the methods used to develop overarching concepts ("synthesised translations")<br>Describe how potential alternative interpretations or explanations were considered in the synthesis<br><br>**Results (page 11) and Discussion (pages 19-26)**

For peer review only

No.	Criteria Headings	Reporting Criteria
Findings		
16	Outcome of synthesis process	Describe the new theory, conceptual framework, model, configuration, or interpretation of data developed from the synthesis<br><br>**Results (third-order constructs) and Discussion (pages 19-26)**
Phase 7—Expressing the synthesis		
Discussion		
17	Summary of findings	Summarize the main interpretive findings of the translation and synthesis and compare them to existing literature<br><br>**Discussion (pages 25-29)**
18	Strengths, limitations, and reflexivity	Reflect on and describe the strengths and limitations of the synthesis:<br>- Methodological aspects—for example, describe how the synthesis findings were influenced by the nature of the included studies and how the meta-ethnography was conducted.- Reflexivity—for example, the impact of the research team on the synthesis findings<br><br>**Discussion, sub-section Strengths and Limitations (pages 27-28)**<br>**Reflexivity (pages 9-10)**
19	Recommendations and conclusions	Describe the implications of the synthesis<br><br>**Discussion (pages 25-29) and Conclusion (page 29)**

For peer review only

Appendix 2: Quality assessment

Study: Calcia MA, Bedi S, Lempp H, Howard LM, Oram S. The healthcare experiences of perpetrators of domestic violence and abuse: a systematic review and meta-synthesis

Assessment based on Harden A, Brunton G, Fletcher A, et al. Teenage pregnancy and social disadvantage: systematic review integrating controlled trials and qualitative studies. *Bmj* 2009;339

Vaan Rooij 2013: 8

Minor concerns over data collection (no mention of pilot interviews)
Minor concerns over analysis methods (no mention of strategies to ensure validity)
No concerns over how the study was conducted (e.g., aims/objectives were clear, sampling methods were clear and appropriate)

Overall assessment: medium

**Quality of reporting**	**Use of strategies to increase reliability and validity**	**Extent to which study findings reflected participants' perspectives and experiences**
Were the **aims and objectives** clearly reported? YES<br>Was there an adequate **description of the context** in which the research was carried out? YES<br>Was there an adequate description of the **sample** and the methods by which the sample was **identified and recruited**? YES<br>Was there an adequate description of the **methods used to collect data**? YES<br>Was there an adequate description of the **methods used to analyse data**? NOT CLEAR (has detailed description of analysis process but does not give name of type of qualitative method)	Were there attempts to establish the **reliability** of the data collection tools (for example, by use of interview topic guides)? YES<br>Were there attempts to establish the **validity** of the data collection tools (for example, with pilot interviews)? NOT CLEAR<br>Were there attempts to establish the **reliability of the data analysis methods** (for example, by use of independent coders)? YES<br>Were there attempts to establish the **validity of data analysis methods** (for example, by searching for negative cases)? NOT CLEAR	Did the study use **appropriate data collection methods** for helping participants to express their views? YES<br>Did the study use appropriate methods for ensuring the data analysis was grounded in the views of participants? YES<br>Did the study actively involve participants in its design and conduct? NOT CLEAR

For peer review only - http://bmjopen.bmj.com/site/about/guidelines.xhtml

Hester 2006 : 5

Minor concerns over data collection
Moderate concerns over analysis methods (method not clear)
Moderate concerns over how the study was conducted (sampling and recruitment methods not clear)

Overall assessment: low

**Quality of reporting**	**Use of strategies to increase reliability and validity**	**Extent to which study findings reflected participants' perspectives and experiences**
Were the aims and objectives clearly reported? YES<br>Was there an adequate description of the context in which the research was carried out? YES<br>Was there an adequate description of the sample and the methods by which the sample was identified and recruited? NO (not clear about how participants were approached or selected)<br>Was there an adequate description of the methods used to collect data? YES<br>Was there an adequate description of the methods used to analyse data? NO	Were there attempts to establish the reliability of the data collection tools (for example, by use of interview topic guides)? YES<br>Were there attempts to establish the validity of the data collection tools (for example, with pilot interviews)? NOT CLEAR<br>Were there attempts to establish the reliability of the data analysis methods (for example, by use of independent coders)? NO<br>Were there attempts to establish the validity of data analysis methods (for example, by searching for negative cases)? NO	Did the study use appropriate data collection methods for helping participants to express their views? YES<br>Did the study use appropriate methods for ensuring the data analysis was grounded in the views of participants? NOT CLEAR<br>Did the study actively involve participants in its design and conduct? NOT CLEAR

Hashimoto 2018: 7
Minor concerns over data collection (no mention of pilot interviews)
Minor concerns over data analysis
No concerns over how study was conducted (aims/objectives, sampling and recruitment were clear and appropriate)

Overall assessment: medium

Quality of reporting	Use of strategies to increase reliability and validity	Extent to which study findings reflected participants' perspectives and experiences
Were the aims and objectives clearly reported? YES<br>Was there an adequate description of the context in which the research was carried out? YES<br>Was there an adequate description of the sample and the methods by which the sample was identified and recruited? YES<br>Was there an adequate description of the methods used to collect data? YES<br>Was there an adequate description of the methods used to analyse data? YES	Were there attempts to establish the reliability of the data collection tools (for example, by use of interview topic guides)? YES<br>Were there attempts to establish the validity of the data collection tools (for example, with pilot interviews)? NOT CLEAR<br>Were there attempts to establish the reliability of the data analysis methods (for example, by use of independent coders)? NOT CLEAR<br>Were there attempts to establish the validity of data analysis methods (for example, by searching for negative cases)? NOT CLEAR	Did the study use appropriate data collection methods for helping participants to express their views? YES<br>Did the study use appropriate methods for ensuring the data analysis was grounded in the views of participants? NOT CLEAR<br>Did the study actively involve participants in its design and conduct? NOT CLEAR

Morgan 2014: 9

Minor concerns over data collection (no mention of pilot interviews)
Minor concerns over data analysis (no mention of strategies to ensure validity)
No concerns over how study was conducted (aims/objectives, sampling and recruitment were clear and appropriate)

Overall assessment: medium

Quality of reporting	Use of strategies to increase reliability and validity	Extent to which study findings reflected participants' perspectives and experiences
Were the aims and objectives clearly reported? YES<br>Was there an adequate description of the context in which the research was carried out? YES<br>Was there an adequate description of the sample and the methods by which the sample was identified and recruited? YES<br>Was there an adequate description of the methods used to collect data? YES<br>Was there an adequate description of the methods used to analyse data? YES	Were there attempts to establish the reliability of the data collection tools (for example, by use of interview topic guides)? YES<br>Were there attempts to establish the validity of the data collection tools (for example, with pilot interviews)? NOT CLEAR<br>Were there attempts to establish the reliability of the data analysis methods (for example, by use of independent coders)? YES<br>Were there attempts to establish the validity of data analysis methods (for example, by searching for negative cases)? NOT CLEAR	Did the study use appropriate data collection methods for helping participants to express their views? YES<br>Did the study use appropriate methods for ensuring the data analysis was grounded in the views of participants? YES<br>Did the study actively involve participants in its design and conduct? NOT CLEAR

Swogger 2016: 9

Minor concerns over data collection (no mention of pilot interviews)
Minor concerns over data analysis (no mention of strategies to ensure validity)
No concerns over how study was conducted (aims/objectives, sampling and recruitment were clear and appropriate)

Overall assessment: medium

**Quality of reporting**	**Use of strategies to increase reliability and validity**	**Extent to which study findings reflected participants' perspectives and experiences**
Were the aims and objectives clearly reported? YES<br>Was there an adequate description of the context in which the research was carried out? YES<br>Was there an adequate description of the sample and the methods by which the sample was identified and recruited? YES<br>Was there an adequate description of the methods used to collect data? YES<br>Was there an adequate description of the methods used to analyse data? YES	Were there attempts to establish the reliability of the data collection tools (for example, by use of interview topic guides)? YES<br>Were there attempts to establish the validity of the data collection tools (for example, with pilot interviews)? NOT CLEAR<br>Were there attempts to establish the reliability of the data analysis methods (for example, by use of independent coders)? YES<br>Were there attempts to establish the validity of data analysis methods (for example, by searching for negative cases)? NOT CLEAR	Did the study use appropriate data collection methods for helping participants to express their views? YES<br>Did the study use appropriate methods for ensuring the data analysis was grounded in the views of participants? YES<br>Did the study actively involve participants in its design and conduct? NOT CLEAR

Bacchus 2018: 7

Minor concerns over data collection (no mention of pilot interviews)
Moderate concerns over data analysis (no mention of strategies to ensure validity)
No concerns over how study was conducted (aims/objectives, sampling and recruitment were clear and appropriate)

Overall assessment: medium

Quality of reporting	Use of strategies to increase reliability and validity	Extent to which study findings reflected participants' perspectives and experiences
Were the aims and objectives clearly reported? YES<br>Was there an adequate description of the context in which the research was carried out? YES<br>Was there an adequate description of the sample and the methods by which the sample was identified and recruited? YES<br>Was there an adequate description of the methods used to collect data? YES<br>Was there an adequate description of the methods used to analyse data? NOT CLEAR	Were there attempts to establish the reliability of the data collection tools (for example, by use of interview topic guides)? YES<br>Were there attempts to establish the validity of the data collection tools (for example, with pilot interviews)? NOT CLEAR<br>Were there attempts to establish the reliability of the data analysis methods (for example, by use of independent coders)? YES<br>Were there attempts to establish the validity of data analysis methods (for example, by searching for negative cases)? NOT CLEAR	Did the study use appropriate data collection methods for helping participants to express their views? YES<br>Did the study use appropriate methods for ensuring the data analysis was grounded in the views of participants? NOT CLEAR<br>Did the study actively involve participants in its design and conduct? NO

Reporting checklist for systematic review and meta-analysis.

Based on the PRISMA guidelines.

Instructions to authors

Complete this checklist by entering the page numbers from your manuscript where readers will find each of the items listed below.

Your article may not currently address all the items on the checklist. Please modify your text to include the missing information. If you are certain that an item does not apply, please write "n/a" and provide a short explanation.

Upload your completed checklist as an extra file when you submit to a journal.

In your methods section, say that you used the PRISMAreporting guidelines, and cite them as:

Moher D, Liberati A, Tetzlaff J, Altman DG, The PRISMA Group. Preferred Reporting Items for Systematic Reviews and Meta-Analyses: The PRISMA Statement

		Reporting Item	Page Number
**Title**			
	#1	Identify the report as a systematic review, meta-analysis, or both.	1, 2
**Abstract**			
Structured summary	#2	Provide a structured summary including, as applicable: background; objectives; data sources; study eligibility criteria, participants, and interventions; study appraisal and synthesis methods; results; limitations; conclusions and implications of key findings; systematic review registration number	2
**Introduction**			
Rationale	#3	Describe the rationale for the review in the context of what is already known.	3

For peer review only

Objectives	#4	Provide an explicit statement of questions being addressed with reference to participants, interventions, comparisons, outcomes, and study design (PICOS).	4, 5
**Methods**			
Protocol and registration	#5	Indicate if a review protocol exists, if and where it can be accessed (e.g., Web address) and, if available, provide registration information including the registration number.	1
Eligibility criteria	#6	Specify study characteristics (e.g., PICOS, length of follow-up) and report characteristics (e.g., years considered, language, publication status) used as criteria for eligibility, giving rational	4, 5
Information sources	#7	Describe all information sources in the search (e.g., databases with dates of coverage, contact with study authors to identify additional studies) and date last searched.	5, 6
Search	#8	Present full electronic search strategy for at least one database, including any limits used, such that it could be repeated.	6
Study selection	#9	State the process for selecting studies (i.e., for screening, for determining eligibility, for inclusion in the systematic review, and, if applicable, for inclusion in the meta-analysis).	6, 7
Data collection process	#10	Describe the method of data extraction from reports (e.g., piloted forms, independently by two reviewers) and any processes for obtaining and confirming data from investigators.	6, 7
Data items	#11	List and define all variables for which data were sought (e.g., PICOS, funding sources), and any assumptions and simplifications made.	7
Risk of bias in individual studies	#12	Describe methods used for assessing risk of bias in individual studies (including specification of whether this was done at the study or outcome level, or both), and how this information is to be used in any data synthesis.	7, 8, 9
Summary measures	#13	State the principal summary measures (e.g., risk ratio, difference in means).	n/a (the study is a meta synthesis of qualitative studies)

For peer review only

Planned methods of analyis	#14	Describe the methods of handling data and combining results of studies, if done, including measures of consistency (e.g., I2) for each meta-analysis.	7
Risk of bias across studies	#15	Specify any assessment of risk of bias that may affect the cumulative evidence (e.g., publication bias, selective reporting within studies).	7, 8, 9
Additional analyses	#16	Describe methods of additional analyses (e.g., sensitivity or subgroup analyses, meta-regression), if done, indicating which were pre-specified.	n/a (the study is a meta synthesis of qualitative studies)

Results

Study selection	#17	Give numbers of studies screened, assessed for eligibility, and included in the review, with reasons for exclusions at each stage, ideally with a flow diagram.	9, 10
Study characteristics	#18	For each study, present characteristics for which data were extracted (e.g., study size, PICOS, follow-up period) and provide the citation.	10-17
Risk of bias within studies	#19	Present data on risk of bias of each study and, if available, any outcome-level assessment (see Item 12).	10, 11
Results of individual studies	#20	For all outcomes considered (benefits and harms), present, for each study: (a) simple summary data for each intervention group and (b) effect estimates and confidence intervals, ideally with a forest plot.	n/a (the study is a meta synthesis of qualitative studies)
Synthesis of results	#21	Present the main results of the review. If meta-analyses are done, include for each, confidence intervals and measures of consistency.	17-19
Risk of bias across studies	#22	Present results of any assessment of risk of bias across studies (see Item 15).	n/a
Additional analysis	#23	Give results of additional analyses, if done (e.g., sensitivity or subgroup analyses, meta-regression [see Item 16]).	n/a (the study is a meta synthesis of qualitative studies)

Discussion

For peer review only

Summary of Evidence	#24	Summarize the main findings, including the strength of evidence for each main outcome; consider their relevance to key groups (e.g., health care providers, users, and policy makers	19-21
Limitations	#25	Discuss limitations at study and outcome level (e.g., risk of bias), and at review level (e.g., incomplete retrieval of identified research, reporting bias).	21
Conclusions	#26	Provide a general interpretation of the results in the context of other evidence, and implications for future research.	21, 22
**Funding**			
Funding	#27	Describe sources of funding or other support (e.g., supply of data) for the systematic review; role of funders for the systematic review.	1

Notes:

- 13: n/a (the study is a meta synthesis of qualitative studies)
- 16: n/a (the study is a meta synthesis of qualitative studies)
- 20: n/a (the study is a meta synthesis of qualitative studies)
- 23: n/a (the study is a meta synthesis of qualitative studies) The PRISMA checklist is distributed under the terms of the Creative Commons Attribution License CC-BY. This checklist was completed on 24. July 2020 using https://www.goodreports.org/, a tool made by the EQUATOR Network in collaboration with Penelope.ai

For peer review only
